# Scattering from Spheres: A New Look into an Old Problem

**DOI:** 10.3390/electronics10020216

**Published:** 2021-01-19

**Authors:** Giuseppe Ruello, Riccardo Lattanzi

**Affiliations:** 1 Department of Electrical Engineering and Information Technology, University of Napoli Federico II, 80125 Naples, Italy; 2 Center for Advanced Imaging Innovation and Research (CAI2R) and Bernard and Irene Schwartz Center for Biomedical Imaging, Department of Radiology, New York University School of Medicine, New York, NY 10016, USA;

**Keywords:** electromagnetic scattering and propagation, Mie scattering, reflection coefficient

## Abstract

In this work, we introduce a theoretical framework to describe the scattering from spheres. In our proposed framework, the total field in the outer medium is decomposed in terms of inward and outward electromagnetic fields, rather than in terms of incident and scattered fields, as in the classical Lorenz–Mie formulation. The fields are expressed as series of spherical harmonics, whose combination weights can be interpreted as reflection and transmission coefficients, which provides an intuitive understanding of the propagation and scattering phenomena. Our formulation extends the previously proposed theory of non-uniform transmission lines by introducing an expression for impedance transfer, which yields a closed-form solution for the fields inside and outside the sphere. The power transmitted in and scattered by the sphere can be also evaluated with a simple closed-form expression and related with the modulus of the reflection coefficient. We showed that our method is fully consistent with the classical Mie scattering theory. We also showed that our method can provide an intuitive physical interpretation of electromagnetic scattering in terms of impedance matching and resonances, and that it is especially useful for the case of inward traveling spherical waves generated by sources surrounding the scatterer.

## 1. Introduction

The study of the electromagnetic scattering from spherical objects has its origin in the work of Lorenz and Mie [1] at the turn of the 20th century. Modern formulations [2–5] of what has since been referred as “Mie scattering” arose from this outstanding work and has since been applied to light scattering, cancer detection, metamaterial theory and much more [6–15].

In Mie scattering, the total electromagnetic (EM) field outside the sphere can be expressed as the sum of the incident field, which is the field that would be there in the absence of the sphere (i.e., the scatterer), and the scattered field. The field that propagates inside the sphere is called transmitted field. The EM fields are expressed as a superposition of vector harmonics. The EM field dependence on each spherical coordinate is factorized, and the radial dependence inside the sphere is defined by means of spherical Bessel functions. To guarantee that the EM field is finite at the origin of the coordinate system, which coincides with the center of the sphere, only spherical Bessel functions of the first kind are used to describe the radial dependence inside the sphere. Outside the sphere, the EM field is typically defined by means of a combination of stationary first kind and progressive fourth kind spherical Bessel functions. Internal and external fields are linked by the continuity conditions.

Despite the elegance and compactness of the Mie analytical formulation, its physical interpretation can be challenging, because both scattered and transmitted field coefficients are expressed as a combination of Bessel and Riccati–Bessel functions. In an effort to improve the physical understanding of the scattering characteristics of spheres, Debye proposed to expand each term of the Mie scattering in series [16–19]. Each term of the Debye series can be interpreted as the result of a diffraction, reflection, or transmission phenomenon at the air–sphere interface. While such formalism improves the physical comprehension, its complexity makes it practical only for a limited number of simple problems.

Building on Mie scattering theory, extensive work has been done in the last decades to provide elegant, closed-form solutions to the problem of scattering from spheres [20,21]. Such work has mainly focused on two types of scattering problems: the case of an outgoing wave incident on a spherical boundary and that of a standing wave incident on a spherical boundary. These problems, well described in [21], have been of great interest because they are useful to analyze the two important applicative fields of scattering theory: the scattering of lights and the irradiation of antennas. In the first case, the incident wave is modeled as a plane wave, whereas in the second case, the source is at the origin and the incident field is an outgoing wave. For example, Mie scattering was developed in the framework of light scattering theory, using plane waves as incident fields and adopting a number of far field approximations for the evaluation of the scattered field. This approach, however, does not help understanding complex near-field behaviors, which are important for biomedical applications.

In fact, in various diagnostic and therapeutic techniques, a local radiofrequency source is used to illuminate a body part, which can be often modeled using a dielectric sphere. Although this third type of scattering problems, in which there is an incoming spherical wave incident on a spherical boundary, is becoming increasingly relevant, current theoretical frameworks only provide limited physical intuition to analyze it. For example, in magnetic resonance imaging (MRI), where the interaction of EM fields with biological tissue affects both image quality and patient safety, dielectric spheres have been used as an approximation of the human head to simulate the performance of radiofrequency coils [22–24]. While rapid analytical approaches based on Mie scattering enable one to explore a large parameter space, they provide limited comprehension of the physical variables that govern the EM field propagation and should guide the design of MRI detectors and transmitters.

An alternative approach that could be more suitable for this type of problems is to express the scattering from the sphere in terms of equivalent transmission lines [25–27]. Schelkunoff, in particular, proposed the theory of the transmission of spherical waves [25]. This outstanding formulation introduced the basic concepts of impedance and reflection coefficient to describe the scattering from a sphere. However, its practical use has been considerably limited, mainly due to the mathematical complexity of the impedances and to the lack of a simple impedance transfer formula. In fact, the latter is critical for simplifying the description and interpretation of boundary condition problems and to relate impedances and reflection coefficients in the case of layered spheres.

This work expands the theory of equivalent transmission lines [25] by providing a closed-form solution to the problem of the scattering of an inward spherical wave on a spherical boundary, which has not been fully addressed by previous work. The main difference with respect to Mie scattering is that both the EM fields outside and inside the scatterer are expressed as a sum of inward (or incident) and outward (or reflected) waves. This field decomposition enables interpreting the ratios between field coefficients as reflection coefficients, providing an intuitive explanation of the physical phenomena that govern the EM field propagation inside the object. In addition, the resulting EM field expressions could be interpreted in terms of non-uniform equivalent transmission lines with the sphere center represented by an equivalent short load. We introduce an impedance transfer formula, which is expected to facilitate the straightforward utilization of our formalism in several applicative scenarios.

The remainder of the paper is organized as follows. In Section 2, the classical Mie scattering is recalled, whereas, in Section 3, the proposed reformulation is described. In Section 4, the two methods are compared. In Section 5, numerical examples are presented to show the advantages of the proposed method for the physical interpretation of phenomena that can be described in the framework of the Mie scattering. The main results are discussed in the concluding session.

## 2. Mie Scattering

The classical Mie scattering formulation is described in several research and review papers [2,4,28] which provide all the physical and mathematical details. In this section, we recall only the basic principles and the equations that will be used as a reference to introduce our approach.

From Maxwell’s equations, the electric field **E** can be expressed as the solution of a Helmholtz equation:

(1) $$\nabla^{2}E+k^{2}E=0$$

where *k* is the wavenumber. An identical equation can be derived for the magnetic field. In the case of scattering from spherical objects, it has been demonstrated [3,4] that the fields can be expressed in terms of the two families of vector harmonics **M**_*nm*_ and **N**_*nm*_:

(2) $$M_{nm}=B_{n}(kr)\left[i\pi_{nm}(\vartheta )\hat{\vartheta }-\tau_{nm}(\vartheta )\hat{\varphi }\right]e^{im\varphi }$$

(3) $$N_{nm}=\frac{1}{kr}\frac{d\left(rB_{n}(kr)\right)}{dr}\left[i\pi_{nm}(\vartheta )\hat{\varphi }+\tau_{nm}(\vartheta )\hat{\vartheta }\right]e^{im\varphi }\;+\frac{B_{n}(kr)}{kr}n(n+1){\text{P}}_{n}^{m}(\text{cos}\;\vartheta )e^{im\varphi_{\hat{r}}}$$

where *i* is the imaginary, (*r*, *ϑ*, *φ*) are the three coordinates of a spherical coordinate system with the origin in the center of the sphere, *B*_*n*_ is a spherical Bessel function of the *n*th order, ${\text{P}}_{n}^{m}$ is the associated Legendre polynomial, *π*_*nm*_(*ϑ*) and *τ*_*nm*_(*ϑ*) are sectorial functions, defined as:

(4) $$\pi_{nm}(\vartheta )=m\frac{{\text{P}}_{n}^{m}(\text{cos}\;\vartheta )}{\text{sin}\;\vartheta }$$

(5) $$\tau_{nm}(\vartheta )=\frac{d{\text{P}}_{n}^{m}(\text{cos}\;\vartheta )}{d\vartheta }$$

The spherical Bessel function *B*_*n*_ is the solution of the Bessel equation in spherical coordinates and can be written as a combination of static first and second kind spherical Bessel functions, or traveling third and fourth kind spherical Bessel functions (also called first and second type spherical Hankel functions). In Mie scattering, the total EM field in the region outside the sphere is expressed as the sum of an incident field:

(6) $${\text{E}}_{i}=\overset{\infty }{\underset{n=1}{\sum }}\overset{n}{\underset{m=-n}{\sum }}\left(a_{nm}M_{nm}^{\left(1\right)}+b_{nm}N_{nm}^{\left(1\right)}\right)\;B_{i}=\frac{k}{i\omega }\overset{\infty }{\underset{n=1}{\sum }}\overset{n}{\underset{m=-n}{\sum }}\left(a_{nm}N_{nm}^{\left(1\right)}+b_{nm}M_{nm}^{\left(1\right)}\right)$$

and a scattered field:

(7) $$E_{s}=\overset{\infty }{\underset{n=1}{\sum }}\overset{n}{\underset{m=-n}{\sum }}\left(c_{nm}M_{nm}^{\left(4\right)}+d_{nm}N_{nm}^{\left(4\right)}\right)\;B_{s}=\frac{k}{i\omega }\overset{\infty }{\underset{n=1}{\sum }}\overset{n}{\underset{m=-n}{\sum }}\left(c_{nm}N_{nm}^{\left(4\right)}+d_{nm}M_{nm}^{\left(4\right)}\right)$$

The field that instead propagates inside the sphere (i.e., the transmitted, field) is described as:

(8) $$E_{t}=\overset{\infty }{\underset{n=1}{\sum }}\overset{n}{\underset{m=-n}{\sum }}\left(e_{nm}M_{nm}^{\left(1\right)}+f_{nm}N_{nm}^{\left(1\right)}\right)\;B_{t}=\frac{k}{i\omega }\overset{\infty }{\underset{n=1}{\sum }}\overset{n}{\underset{m=-n}{\sum }}\left(e_{nm}N_{nm}^{\left(1\right)}+f_{nm}M_{nm}^{\left(1\right)}\right)$$

The superscripts ^(1)^ and ^(4)^ appended to the vector harmonics in Equations (6)–(8) indicate the kind of the spherical Bessel functions used for the description of the radial dependence. Since the incident and the transmitted fields are defined at the origin, their expressions include only spherical Bessel functions of the first kind, which impose that the field is finite at the origin. The scattered field is instead outward directed, so it is described as a superposition of spherical Bessel functions of the fourth kind (also called spherical Hankel functions of the second kind). Note that this notation is based on a *e*^*iωt*^ time dependence. If the *e*^*−iωt*^ notation is used, the outward waves must be described by spherical Bessel functions of the third kind.

Since the **M**_*nm*_ and **N**_*nm*_ vectors are orthogonal and, as shown by Equations (2) and (3), the **M**_*nm*_ vectors have no radial component, the evaluation of the field coefficients in Equations (6)–(8) can be separated in two independent problems. If all the *b*_*nm*_ coefficients are null, the electric field is orthogonal to the radial direction and the solution is called Transverse Electric (TE); if all the *a*_*nm*_ coefficients are null, the magnetic field is orthogonal to the radial direction and the solution is called Transverse Magnetic (TM).

The Mie scattering coefficients are obtained by imposing the continuity of the tangential components of the field at the sphere boundary. The complete Mie formulation is available in the literature. Here, we recall only the expressions for the scattering coefficients, which will be used as a reference to evaluate our method and can be expressed as a combination of spherical Bessel functions:

(9) $$c_{nm}=-a_{nm}\frac{j_{n}^{\prime }\left(k_{1}a\right)j_{n}\left(k_{2}a\right)-\chi j_{n}\left(k_{1}a\right)j_{n}^{\prime }\left(k_{2}a\right)}{h_{n}^{(2{)}^{\prime }}\left(k_{1}a\right)j_{n}\left(k_{2}a\right)-\chi h_{n}^{\left(2\right)}\left(k_{1}a\right)j_{n}^{\prime }\left(k_{2}a\right)}\;d_{nm}=-b_{nm}\frac{j_{n}\left(k_{1}a\right)j_{n}^{\prime }\left(k_{2}a\right)-\chi j_{n}^{\prime }\left(k_{1}a\right)j_{n}\left(k_{2}a\right)}{h_{n}^{\left(2\right)}\left(k_{1}a\right)j_{n}^{\prime }\left(k_{2}a\right)-\chi h_{n}^{(2{)}^{\prime }}\left(k_{1}a\right)j_{n}\left(k_{2}a\right)}$$

where *a* is the radius of the sphere, $\chi =\frac{k_{2}}{k_{1}}$ is the refraction index, *k*_1_ and *k*_2_ are the wavenumbers of the external and internal medium, respectively, and the following definition for the first derivative of the spherical Bessel functions was used:

(10) $$B_{n}^{\prime }(kr)=\frac{1}{kr}\frac{d\left(rB_{n}(kr)\right)}{dr}$$

For a perfectly conducting sphere, the coefficients reduce to:

(11) $$c_{nm}=-a_{nm}\frac{j_{n}\left(k_{1}a\right)}{h_{n}^{\left(2\right)}\left(k_{1}a\right)}\;d_{nm}=-b_{nm}\frac{j_{n}^{\prime }\left(k_{1}a\right)}{h_{n}^{(2{)}^{\prime }}\left(k_{1}a\right)}$$

The above equations represent an outstanding contribution to modern physics, which inspired several works in different fields of science [1,28]. However, their physical interpretation is not immediate.

## 3. Proposed Method

We propose a new formulation of the scattering by spheres, in which the EM field is expressed in terms of inward and outward waves rather than incident, transmitted and scattering waves. Our method, which builds on previous work on equivalent transmission lines [20,21,25], develops from the physical consideration that the inward waves focus in the origin and the energy they carry is redistributed by the outward waves. In other words, the origin can be seen as a sink for the incoming waves and a source of outgoing waves, in accordance with the energy conservation principle.

From the mathematical point of view, in the classical Mie scattering, the energy conservation principle is respected by forcing the radial dependence of the EM field to behave as spherical Bessel functions of the first kind, guaranteeing that the fields are finite in the origin. In our approach, we keep the distinction between inward (described by Hankel functions of the first kind) and outward (described by Hankel functions of the second kind) waves and we fulfill the energy conservation principle by forcing the equality of their field coefficients inside the sphere. From the physics point of view, this constraint is nothing but an energy conservation criterion and it is coherent with the fact that inside the sphere the field behaves as a first kind spherical Bessel function, as described by the Mie scattering. In this framework, the outward waves can be viewed as the result of a reflection phenomenon happening at the origin and the scattering problem can be described by the non-uniform transmission line theory [29], with the origin acting as a perfect reflector.

In the following paragraphs, we describe the proposed formulation for the TE case. The TM solution can be calculated with an analogous procedure.

### 3.1. Problem Formulation

**Figure 1.**
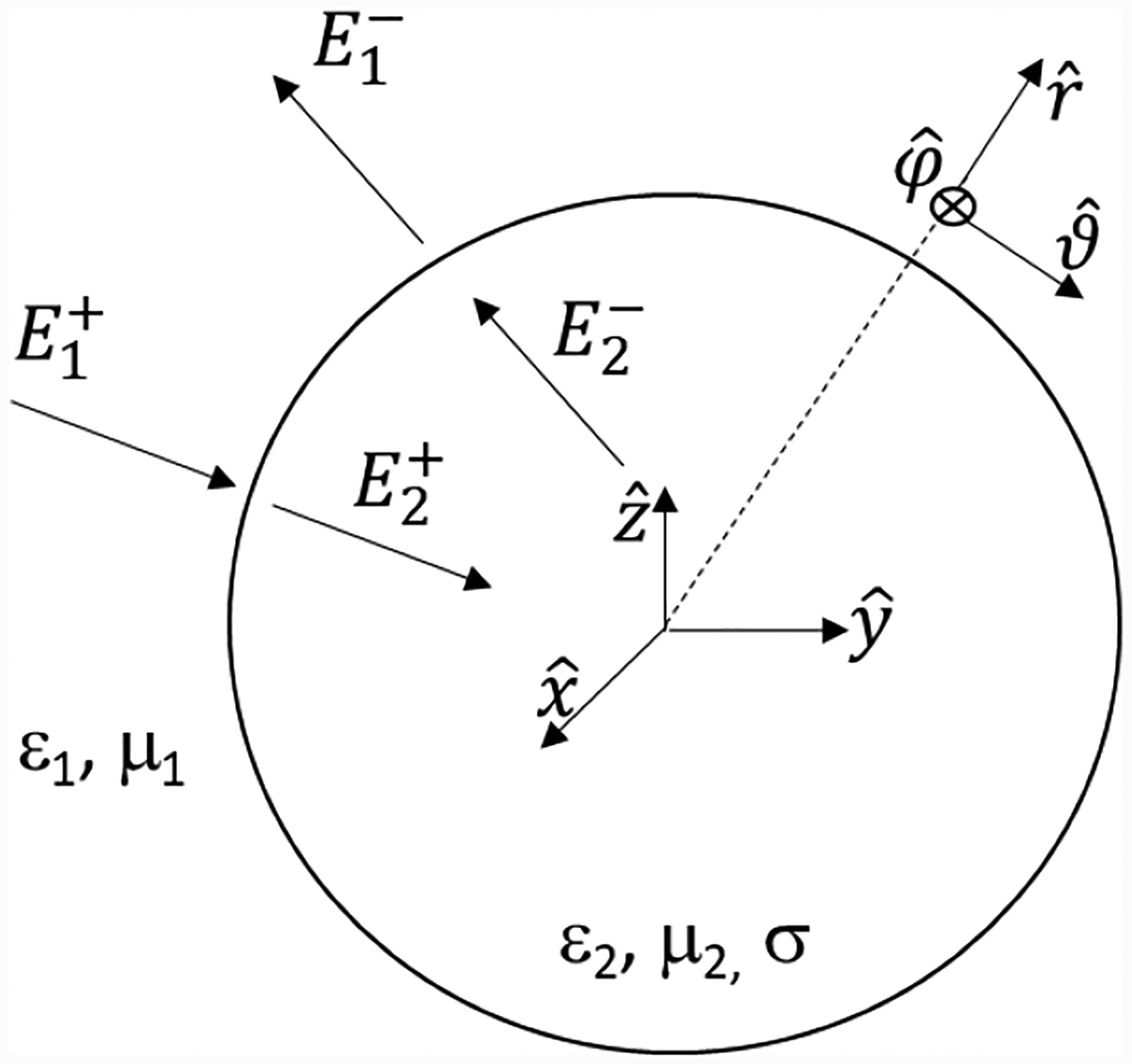
Geometrical representation of the scattering problem. The field in each medium is expressed as the combination of inward and outward spherical waves.

Figure 1 shows a schematic representation of the scattering problem. In Medium 1 (usually air), the total field can be expressed as a superposition of inward and outward waves (note that no distinction is made between incident and scattered field):

(12) $$E_{1}(r)=\overset{\infty }{\underset{n=1}{\sum }}\overset{n}{\underset{m=-n}{\sum }}E_{1nm}^{+}M_{nm}^{\left(3\right)}+E_{1nm}^{-}M_{nm}^{\left(4\right)}\;H_{1}(r)=\frac{k}{i\omega \mu }\overset{\infty }{\underset{n=1}{\sum }}\overset{n}{\underset{m=-n}{\sum }}E_{1nm}^{+}N_{nm}^{\left(3\right)}+E_{1nm}^{-}M_{nm}^{\left(4\right)}$$

In Medium 2 (i.e., inside the sphere), the fields can also be expressed as a superposition of inward and outward waves:

(13) $$E_{2}(r)=\overset{\infty }{\underset{n=1}{\sum }}\overset{n}{\underset{m=-n}{\sum }}E_{2nm}^{+}M_{nm}^{\left(3\right)}+E_{2nm}^{-}M_{nm}^{\left(4\right)}\;H_{2}(r)=\frac{k}{i\omega \mu }\overset{\infty }{\underset{n=1}{\sum }}\overset{n}{\underset{m=-n}{\sum }}E_{2nm}^{+}N_{nm}^{\left(3\right)}+E_{2nm}^{-}N_{nm}^{\left(4\right)}$$

with the constraint that the inside the sphere inward and outward coefficients are equal $\left(E_{2nm}^{+}=E_{2nm}^{-}\right)$ to ensure energy conservation.

The fields **E**_1_(*r*) and **E**_2_(*r*) are linked by the continuity conditions, which allow one to calculate the coefficients of the series expansion.

### 3.2. Characteristic Impedance

We can define an impedance term by taking the ratio between the tangential component of electric and magnetic fields. From the definition of **M**_*nm*_ and **N**_*nm*_ in Equations (2) and (3), for a single wave, the impedance can be expressed as:

(14) $$Z_{n}\left(k_{l}r\right)=\frac{i\omega \mu }{k_{l}}\frac{B_{n}\left(k_{l}r\right)}{B_{n}^{\prime }\left(k_{l}r\right)}$$

where the term $B_{n}^{\prime }\left(k_{l}r\right)$ is defined in Equation (10), *l* ∈ (1, 2) specifies the medium and *n* is the order of the Bessel function.

By observing that the ratio $\frac{\omega \mu }{k_{l}}=\zeta_{l}$ is the characteristic impedance of the *l*-th medium and using the logarithmic derivative of the Riccati–Bessel function:

(15) $$\frac{B_{n}^{\prime }\left(k_{l}r\right)}{B_{n}\left(k_{l}r\right)}=D_{nl}$$

the impedance can be written in a more compact form as $Z_{n}\left(k_{l}r\right)=i\frac{\zeta_{l}}{D_{nl}}$.

The impedances for the inward and outward waves are different, but their values are closely related. In fact, we can evaluate the impedance of the inward wave $Z_{n}^{\left(1\right)}\left(k_{l}r\right)$, by substituting $h_{n}^{\left(1\right)}\left(k_{l}r\right)$ in place of *B*_*n*_(*k*_*l*_*r*) in Equation (14), obtaining:

(16) $$Z_{n}^{\left(1\right)}\left(k_{l}r\right)=\frac{i\omega \mu }{k_{l}}\frac{h_{n}^{\left(1\right)}\left(k_{l}r\right)}{h_{n}^{(1)\prime }\left(k_{1}r\right)}=Z_{nl}$$

whereas we can evaluate the impedance of the outward wave $Z_{n}^{\left(2\right)}\left(k_{l}r\right)$ by substituting $h_{n}^{\left(2\right)}\left(k_{l}r\right)$ in place of *B*_*n*_(*k*_*l*_*r*) in Equation (14), obtaining:

(17) $$Z_{n}^{\left(2\right)}\left(k_{l}r\right)=\frac{i\omega \mu }{k_{l}}\frac{h_{n}^{\left(2\right)}\left(k_{l}r\right)}{h_{n}^{(2)\prime }\left(k_{l}r\right)}=Z_{n}^{\left(1\right)}\left(-k_{l}r\right)=\bar{Z_{nl}}$$

Inside the sphere, given the energy conservation constraint $E_{2nm}^{+}=E_{2nm}^{-}$, and given the identity $h_{n}^{\left(1\right)}\left(k_{2}r\right)+h_{n}^{\left(2\right)}\left(k_{2}r\right)=2j_{n}\left(k_{2}r\right)$, the total field (inward plus outward wave) behaves as a spherical Bessel function of the first kind. Therefore, by substituting *j*_*n*_(*k*_2_*r*) in place of *B*_*n*_(*k*_*l*_*r*) in Equation (14), we can define the impedance of the total field inside the sphere as:

(18) $$Z_{n}^{\left(J\right)}\left(k_{2}r\right)=\frac{i\omega \mu }{k_{2}}\frac{j_{n}\left(k_{2}r\right)}{j_{n}^{\prime }\left(k_{2}r\right)}=Z_{Jn2}$$

In Table 1, the different expressions (16)–(18) that Equation (14) can take for different Bessel functions are provided. In the last column, a compact expression that will be used in the remainder of the paper is introduced.

### 3.3. Field Expression: Traveling Form

For each mode of the EM field in the *l*-th medium, the tangential fields can be expressed as:

$$E_{lnm}(r)=E_{lnm}^{+}h_{n}^{\left(1\right)}\left(k_{l}r\right)+E_{lnm}^{-}h_{n}^{\left(2\right)}\left(k_{l}r\right)$$

(19) $$H_{lnm}(r)=\frac{k_{l}}{i\omega \mu }\left[E_{lnm}^{+}h_{n}^{(1{)}^{\prime }}\left(k_{l}r\right)+E_{lnm}^{-}h_{n}^{(2{)}^{\prime }}\left(k_{l}r\right)\right]\;=\left[\frac{E_{lnm}^{+}}{Z_{nl}}h_{n}^{\left(1\right)}\left(k_{l}r\right)+\frac{E_{lnm}^{-}}{\bar{Z_{nl}}}h_{n}^{\left(2\right)}\left(k_{l}r\right)\right]$$

If we define a reflection coefficient for the electric field as:

(20) $$\Gamma_{n}\left(k_{l}r\right)=\frac{E_{l}^{-}h_{nm}^{\left(2\right)}\left(k_{l}r\right)}{E_{l}^{+}h_{nm}^{\left(1\right)}\left(k_{l}r\right)},$$

we can write the fields in a more compact form:

(21) $$E_{lnm}(r)=E_{lnm}^{+}h_{n}^{\left(1\right)}\left(k_{l}r\right)\left[1+\Gamma_{n}\left(k_{l}r\right)\right]\;H_{lnm}(r)=\frac{E_{lnm}^{+}}{Z_{nl}}h_{n}^{\left(1\right)}\left(k_{l}r\right)\left[1+\Gamma_{n}\left(k_{l}r\right)\frac{Z_{nl}}{\bar{Z_{nl}}}\right].$$

The impedance of the total field can then be expressed as a function of the reflection coefficient by taking the ratio between the electric and magnetic field in Equations (21):

(22) $$Z_{n}\left(k_{l}r\right)=\frac{E_{l}(r)}{H_{l}(r)}=Z_{nl}\frac{1+\Gamma_{n}\left(k_{l}r\right)}{1+\Gamma_{n}\left(k_{l}r\right)\frac{Z_{nl}}{\bar{Z_{nl}}}}.$$

Inside the sphere (*l* = 2), this corresponds to the impedance in Equation (18). The above expression can be easily inverted in order to express the reflection coefficients in terms of impedance as:

(23) $$\Gamma_{n}\left(k_{l}r\right)=\frac{Z_{n}\left(k_{l}r\right)-Z_{nl}}{Z_{nl}-Z_{n}\left(k_{l}r\right)\frac{Z_{nl}}{\bar{Z_{nl}}}}$$

The last equations provide a framework for the physical interpretation of the fields. For example, in Equations (21), the electric field is expressed as the product of the term 1 + Γ_*n*_(*k*_*l*_*r*), which accounts for the coherent sum of incident and reflected fields, with the term $h_{n}^{\left(1\right)}\left(k_{l}r\right)$, which accounts for the radial distribution of the energy. One advantage of the proposed approach compared to the classical Mie formulation is that the reflection coefficient and the impedance are scalar and physically interpretable engineering quantities, as opposed to the coefficients in Equation (9). For instance, impedance and reflection coefficient enable to easily evaluate, for each mode, the position of the peak of the electric field inside the sphere, providing a powerful tool for, e.g., antenna design optimization. In Section V, numerical examples are presented to illustrate applications of the proposed methods.

In the equation for the magnetic field (21), the reflection coefficient of the electric field is multiplied by the factor ${\text{Z}}_{nl}/\bar{{\text{Z}}_{nl}}$, which is a term with unitary amplitude. At the origin, the phase of this term is null; therefore, the magnetic reflection coefficient is equal to the electric reflection coefficient.

**Figure 2.**
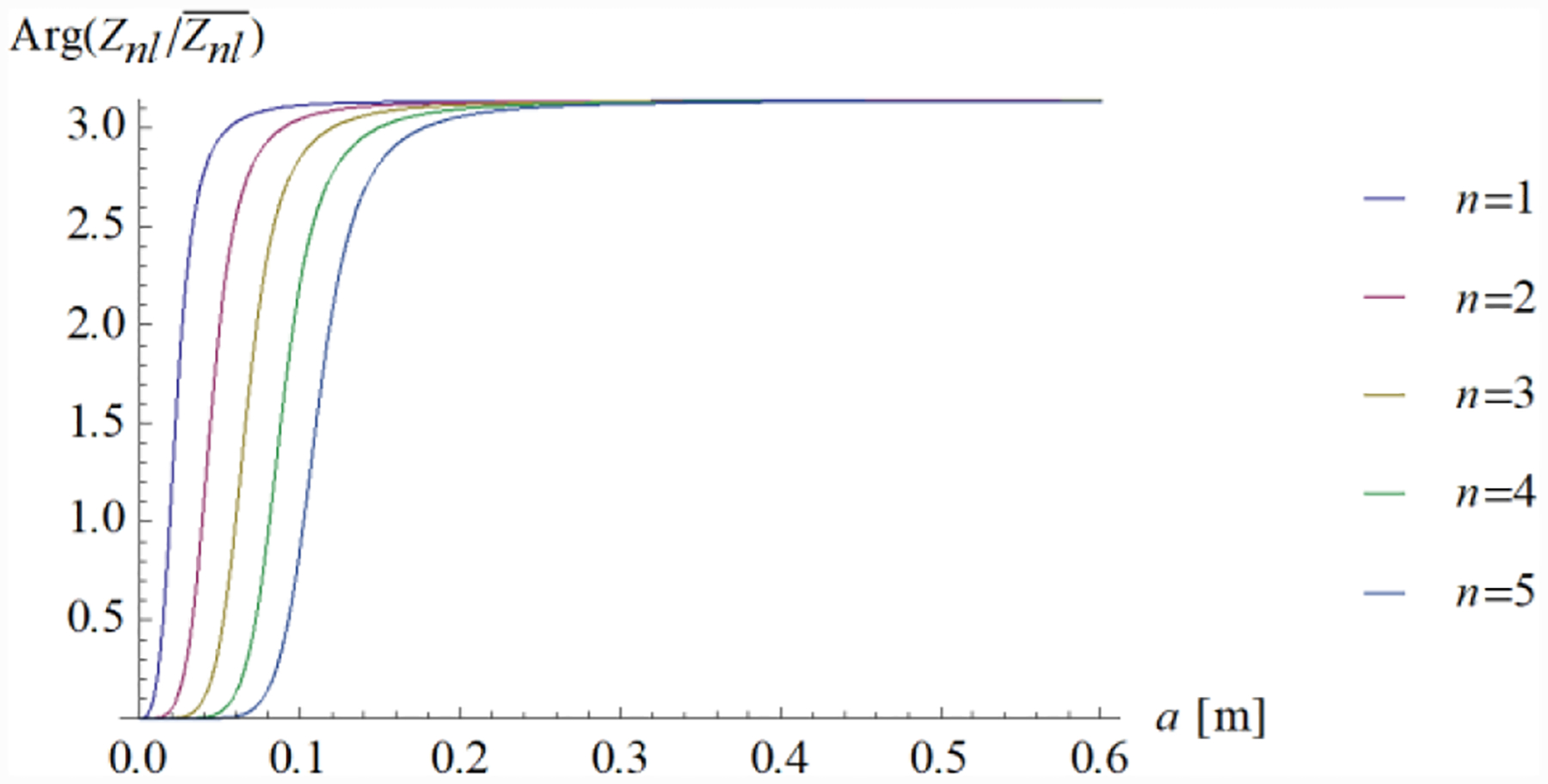
The phase of the term ${\text{Z}}_{nl}/\bar{{\text{Z}}_{nl}}$ is plotted against the sphere radius *a*. The frequency is 297.2 MHz; the sphere dielectric properties are: *ε* = 50*ε*_0_, *σ* = 0; the corresponding wavenumber *k* = 44.89 m^−1^.

For *k*_*l*_ *>> n*, the phase of ${\text{Z}}_{nl}/\bar{{\text{Z}}_{nl}}$ approaches *π*, and the reflection coefficient of the magnetic field is the opposite of that of the electric field, as it happens for transmission lines. The phase of ${\text{Z}}_{nl}/\bar{{\text{Z}}_{nl}}$ is plotted against the sphere radius in Figure 2.

### 3.4. Field Expression: Stationary Form

The spherical Bessel functions of the third and fourth kind can be expressed in terms of stationary spherical Bessel functions as $h_{n}^{\left(1\right)}\left(k_{l}r\right)=j_{n}\left(k_{l}r\right)+iy_{n}\left(k_{l}r\right)$ and $h_{n}^{\left(2\right)}\left(k_{l}r\right)=j_{n}\left(k_{l}r\right)-iy_{n}\left(k_{l}r\right)$. Then, the EM field can be written in stationary form as:

(24) $$E_{lnm}(r)=\left(E_{lnm}^{+}+E_{lnm}^{-}\right)2j_{n}\left(k_{l}r\right)+i\left(E_{lnm}^{+}-E_{lnm}^{-}\right)2y_{n}\left(k_{l}r\right)\;H_{lnm}(r)=\frac{k_{l}}{i\omega \mu }\left(E_{lnm}^{+}+E_{lnm}^{-}\right)2j_{n}^{\prime }\left(k_{l}r\right)+i\left(E_{lnm}^{+}-E_{lnm}^{-}\right)2iy_{n}^{\prime }\left(k_{l}r\right)$$

The corresponding impedance in the *l*-th medium can be calculated, for each mode, as the ratio between electric and magnetic fields:

(25) $$Z_{n}\left(k_{l}r\right)=\frac{E_{l}(r)}{H_{l}(r)}=Z_{Jnl}\frac{A_{0l}+it_{nl}}{A_{0l}+it_{nl}^{\prime }}$$

where

(26) $$A_{0l}=\frac{\left(E_{lnm}^{+}+E_{lnm}^{-}\right)}{\left(E_{lnm}^{+}-E_{lnm}^{-}\right)}$$

(27) $$t_{nl}=\frac{y_{n}\left(k_{l}r\right)}{j_{n}\left(k_{l}r\right)},$$

and

(28) $$t_{nl}^{\prime }=\frac{y_{n}^{\prime }\left(k_{l}r\right)}{j_{n}^{\prime }\left(k_{l}r\right)}$$

Equation (25) provides a novel general expression of the impedance in any medium and it is particularly useful for the evaluation of the matching condition. For example, in case of scattering from multi-layered spheres, it provides an operative expression to evaluate the impedance in any layer as a transfer of the impedance in the origin.

**Figure 3.**
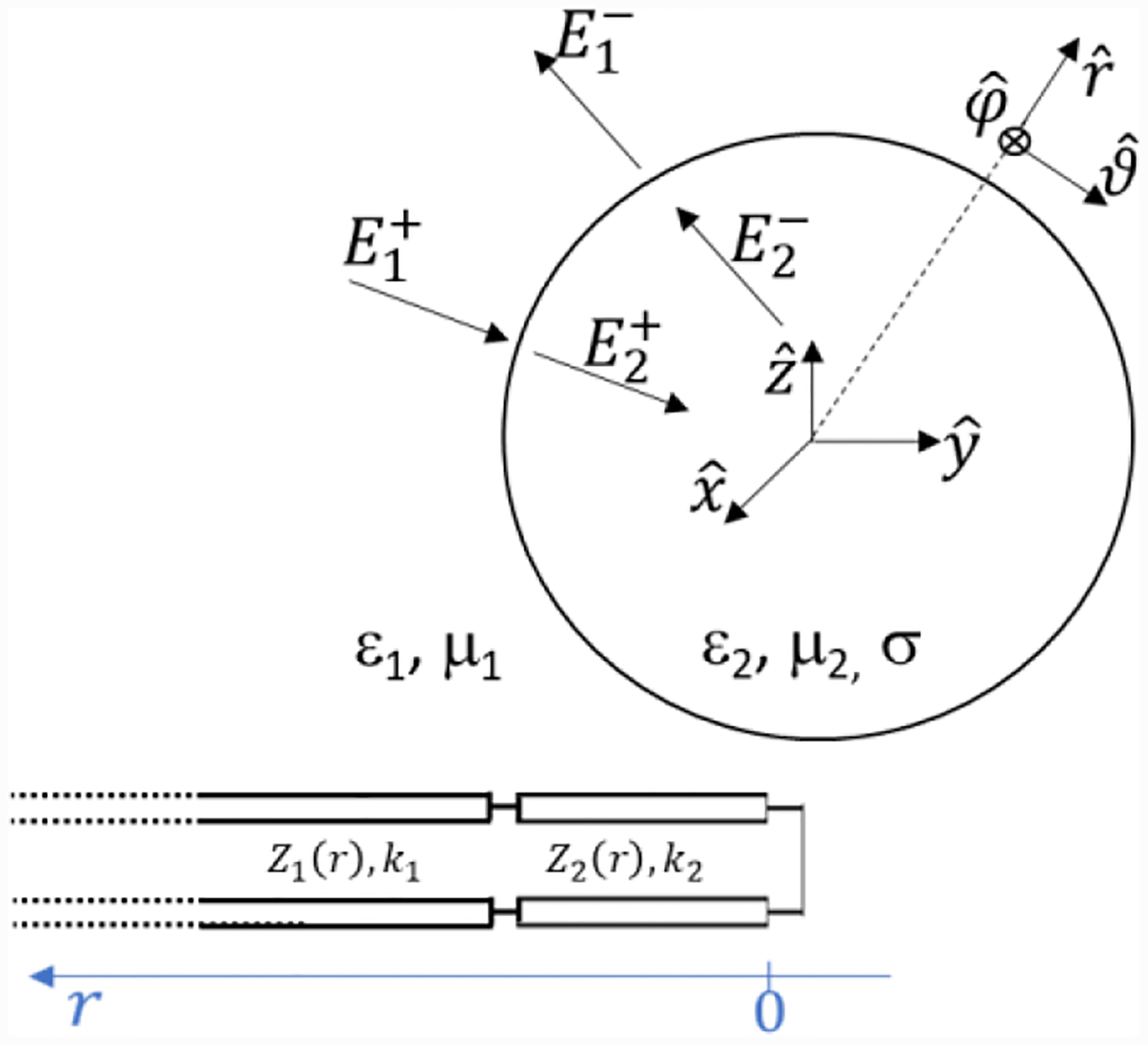
Equivalent transmission line for the case of an inward spherical wave incident on a spherical boundary.

As a proof of consistence with Mie scattering, we can see that, inside the sphere, given the condition $E_{2nm}^{+}=E_{2nm}^{-}$, we get that *A*_02_ = ∞ and the impedence reduces to the *Z*_*Jn*2_ in Equation (18). The importance of Equation (25) is further demonstrated by the fact that, at the origin, *Z*_*Jn*2_ = 0 and this allows interpreting the scattering in terms of equivalent transmission lines closed on a short circuit at the origin, as shown in Figure 3.

### 3.5. Field Coefficients Evaluation

In some applications (for example, when modeling an MRI experiment) an inward wave is generated in the outer medium (by an antenna) and impinges on the sphere. Therefore, it is of interest to express the first medium outward wave ($E_{1nm}^{-}$) and the internal inward wave ($E_{2nm}^{+}$) field coefficient as a function of the first medium inward wave ($E_{1nm}^{+}$) field coefficient. To this purpose, we can use the reflection coefficient definition and the continuity of the impedance. In fact, by inverting Equation (20) and evaluating it at *r = a*, we obtain:

(29) $$E_{1nm}^{-}=E_{1nm}^{+}\Gamma_{n}\left(k_{1}a\right)\frac{h_{n}^{\left(1\right)}\left(k_{1}a\right)}{h_{n}^{\left(2\right)}\left(k_{1}a\right)}$$

The reflection coefficient can be written in terms of the impedance:

(30) $$\Gamma_{n}\left(k_{1}a\right)=\frac{Z_{n}\left(k_{1}a\right)-Z_{n1}}{Z_{n1}-Z_{n}\left(k_{1}a\right)R}$$

where *R* is the ratio $Z_{nl}/\bar{Z_{ni}}$, evaluated in *r* = *a*. By imposing the continuity condition for the impedance in *r* = *a*: *Z*_*n*_ (*k*_1_*a*) = *Z*_*n*_ (*k*_2_*a*) = *Z*_*Jn*2_ (*k*_2_*a*), we can calculate the outward wave coefficients in terms of speherical Bessel functions as:

(31) $$\frac{E_{1nm}^{-}}{E_{1nm}^{+}}=\frac{Z_{Jn2}\left(k_{2}a\right)-Z_{n1}}{Z_{n1}-Z_{Jn2}\left(k_{2}a\right)R}\cdot \frac{h_{n}^{\left(1\right)}\left(k_{1}a\right)}{h_{n}^{\left(2\right)}\left(k_{1}a\right)}$$

The previous expression is the product of two ratios. The first ratio describes a reflection coefficient, whose value depends on the dielectric properties of the media and the radius of the sphere. The second ratio represents a propagation factor, which accounts for the spatial distribution of the energy and whose value also depends on the radius of the sphere.

The coefficients of the field transmitted in the sphere (13) are evaluated by imposing the continuity of the electric field at *r* = *a*. From Equation (21), we have:

(32) $$E_{2nm}^{+}=E_{1nm}^{+}\frac{h_{n}^{\left(1\right)}\left(k_{1}a\right)}{h_{n}^{\left(1\right)}\left(k_{2}a\right)}\frac{\left[1+\Gamma_{n}\left(k_{1}a\right)\right]}{\left[1+\Gamma_{n}\left(k_{2}a\right)\right]}.$$

An expression for reflection coefficient Γ_*n*_(*k*_2_*a*) can be obtained from Equation (20), with the physical constraint $E_{2nm}^{+}=E_{2nm}^{-}$, from being inside the sphere, and substituted in Equation (32):

(33) $$E_{2nm}^{+}=E_{1nm}^{+}\frac{h_{n}^{\left(1\right)}\left(k_{1}a\right)}{2j_{n}\left(k_{2}a\right)}\left[1+\Gamma_{n}\left(k_{1}a\right)\right]$$

The expression of the coefficient has a straightforward physical interpretation also in this case. It is the product of a propagation term that accounts for the geometry-dependent energy distribution and a transmission coefficient τ_*n*_ = 1 + Γ_*n*_(*k*_1_*a*), which accounts for the energy propagated inside the sphere.

### 3.6. Power Evaluation

The traveling form presented in Section 3.3 is useful to describe the field inside the sphere as the coherent sum of inward and outward waves. It also allows one to evaluate the power density *S*_*lnm*_ as:

(34) $$S_{lnm}=\frac{1}{2}E_{lnm}H_{lnm}^{*}\cdot \hat{r}=\;=\frac{1}{2Z_{nl}}{\left|E_{lnm}^{+}\right|}^{2}{\left|h_{n}^{\left(1\right)}\left(k_{l}r\right)\right|}^{2}\left[1+\Gamma_{n}\left(k_{l}r\right)\right]{\left[1+\Gamma_{n}\left(k_{l}r\right)\frac{Z_{nl}}{\bar{Z_{ni}}}\right]}^{*}$$

where $\hat{r}$ is the radial unit vector defined in Figure 1.

When *kr >> n*, the magnetic field is the opposite of the electric field and the power density in each medium can be written as:

(35) $$S_{lnm}=\frac{1}{2Z_{nl}}{\left|E_{lnm}^{+}\right|}^{2}{\left|h_{n}^{\left(1\right)}\left(k_{l}r\right)\right|}^{2}\left[1-{\left|\Gamma_{n}\left(k_{l}r\right)\right|}^{2}\right].$$

This expression clearly shows that the inward and outward powers are decoupled. By integrating the power density on a spherical surface centered at the origin, the power dissipated inside the sphere by the *n*-th mode can be calculated in a straightforward manner by substituting *l* = 2 in Equation (34) and multiplying the result with *n*(*n* + 1)*r*^2^.

In conclusion, in Table 2, the formulas of electromagnetic fields, reflection coefficient and impedances proposed in both traveling and stationary forms are presented.

## 4. Comparison with Mie Scattering

In this section, we compare the proposed framework with the classical Mie scattering. In our approach, the electric field in the external medium *E*_1*nm*_ is expressed as the sum of inward and outward waves:

(36) $$E_{1nm}=E_{1nm}^{+}h_{n}^{\left(1\right)}\left(k_{1}r\right)+E_{1nm}^{-}h_{n}^{\left(2\right)}\left(k_{1}r\right)$$

As both the incident and the scattered fields contribute to the outward waves, we can rewrite Equation (36) to explicitly show both contributions:

(37) $$E_{1nm}=E_{1nm}^{+}h_{n}^{\left(1\right)}\left(k_{1}r\right)+E_{1nm}^{+}h_{n}^{\left(2\right)}\left(k_{1}r\right)+\left(E_{1nm}^{-}-E_{1nm}^{+}\right)h_{n}^{\left(2\right)}\left(k_{1}r\right)$$

The first two terms of Equation (37) represent the incident field, and the last term is the scattering term, as defined in the Mie formulation. Therefore, considering that $h_{n}^{\left(1\right)}\left(k_{1}r\right)+h_{n}^{\left(2\right)}\left(k_{1}r\right)=2j_{n}\left(k_{1}r\right)$, the relationship between our coefficients and those of the classical Mie scattering is:

(38) $$E_{1nm}^{+}=\frac{a_{nm}}{2}$$

(39) $$E_{1nm}^{-}-E_{1nm}^{+}=c_{nm}$$

Therefore, the Mie coefficients can be easily retrieved from the coefficients of our method as:

(40) $$\frac{c_{nm}}{a_{nm}}=\frac{1}{2}\left(\frac{E_{1nm}^{-}}{E_{1nm}^{+}}-1\right)$$

As a further validation of our approach, in Appendix A we present the algebraic passages to obtain the *c*_*nm*_ expression presented in Equation (9) from Equation (40).

The consistence of the proposed model with Mie scattering is directly verified for the case of a perfectly conducting sphere. In fact, the continuity conditions dictate that the electric field at *r* = *a* must be null. This means that the reflection coefficient of the electric field must be −1.

Therefore, the relation between the outward and inward coefficients is immediately found from Equation (29) as:

(41) $$E_{inm}^{-}=-E_{inm}^{+}\frac{h_{n}^{\left(1\right)}\left(k_{1}a\right)}{h_{n}^{\left(2\right)}\left(k_{1}a\right)}$$

Substituting the last equation in Equation (40), we obtain:

(42) $$\frac{c_{nm}}{a_{nm}}=\frac{1}{2}\left(\frac{h_{n}^{\left(1\right)}\left(k_{1}a\right)}{h_{n}^{\left(2\right)}\left(k_{1}a\right)}-1\right)=-\frac{j_{n}\left(k_{1}a\right)}{h_{n}^{\left(2\right)}\left(k_{1}a\right)},$$

which is, in fact, the expression of the Mie scattering coefficients for the perfectly conducting sphere reported in Equation (11).

## 5. Numerical Results

In this section, we present examples of EM scattering from a spherical object and show how our model can provide a physically intuitive understanding of the results.

### 5.1. Results for the Fundamental Mode (n = 1)

We first investigated the scattering and propagation characteristics for the fundamental mode (*n* = 1) as a function of the dielectric properties of the sphere.

**Figure 4.**
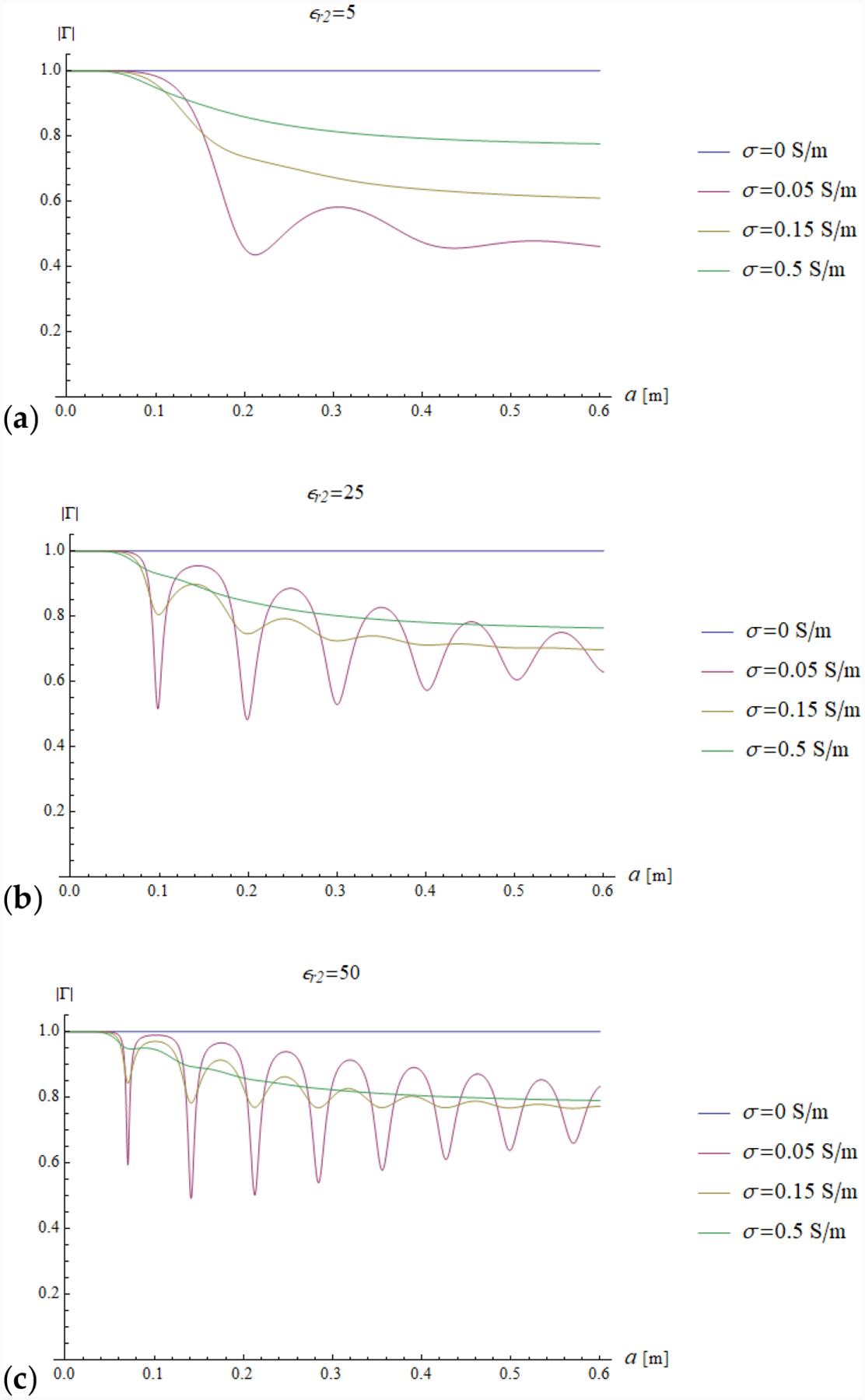
The modulus of the reflection coefficient is plotted as a function of the sphere radius for different values of relative permittivity (*ε*_*r*2_ = 5, 25 and 50 in (**a–c**), respectively) and conductivity (*σ* = 0, 0.05, 0.15 and 0.5 S/m). The frequency is set to 297.2 MHz.

**Figure 5.**
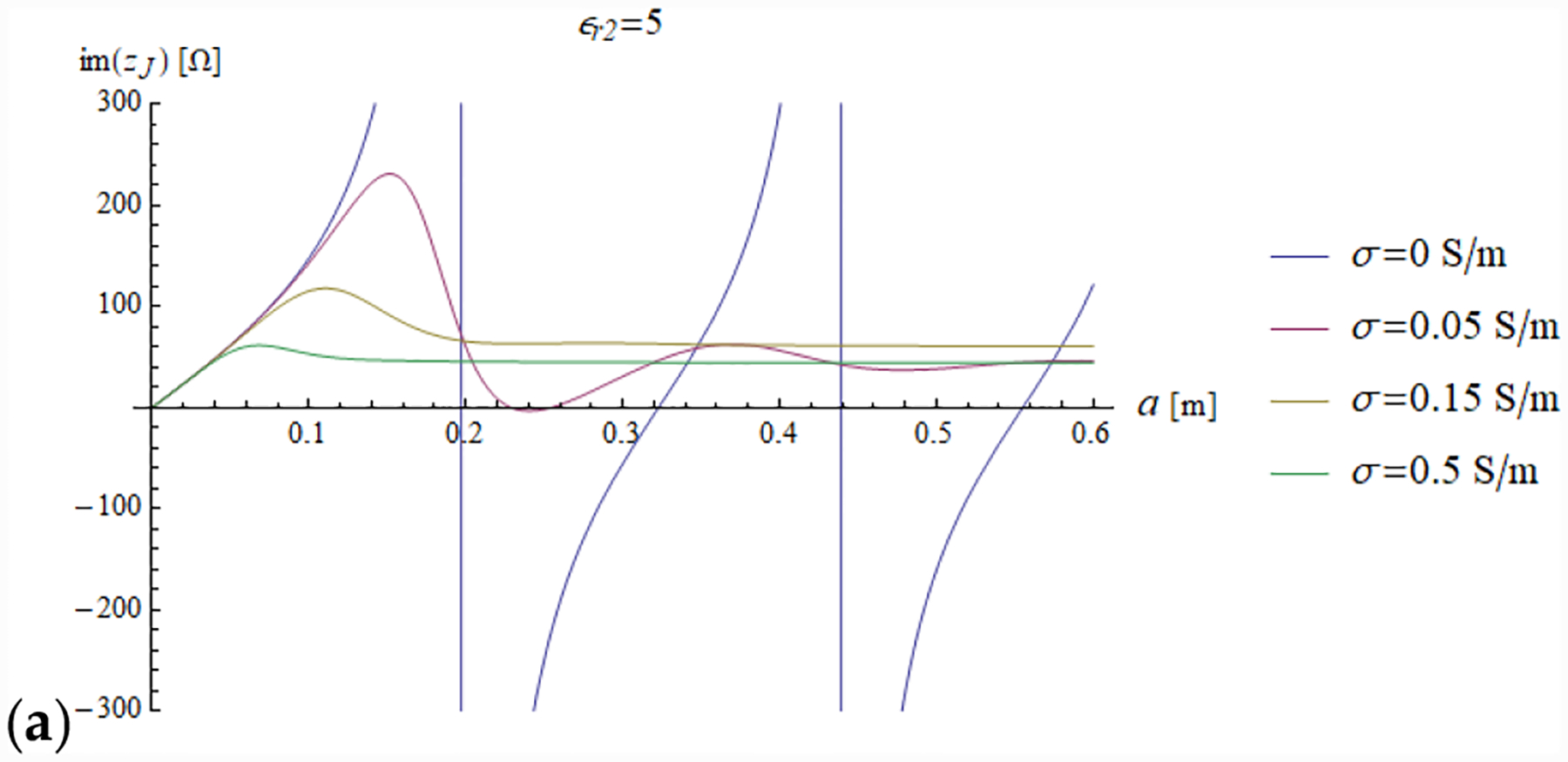

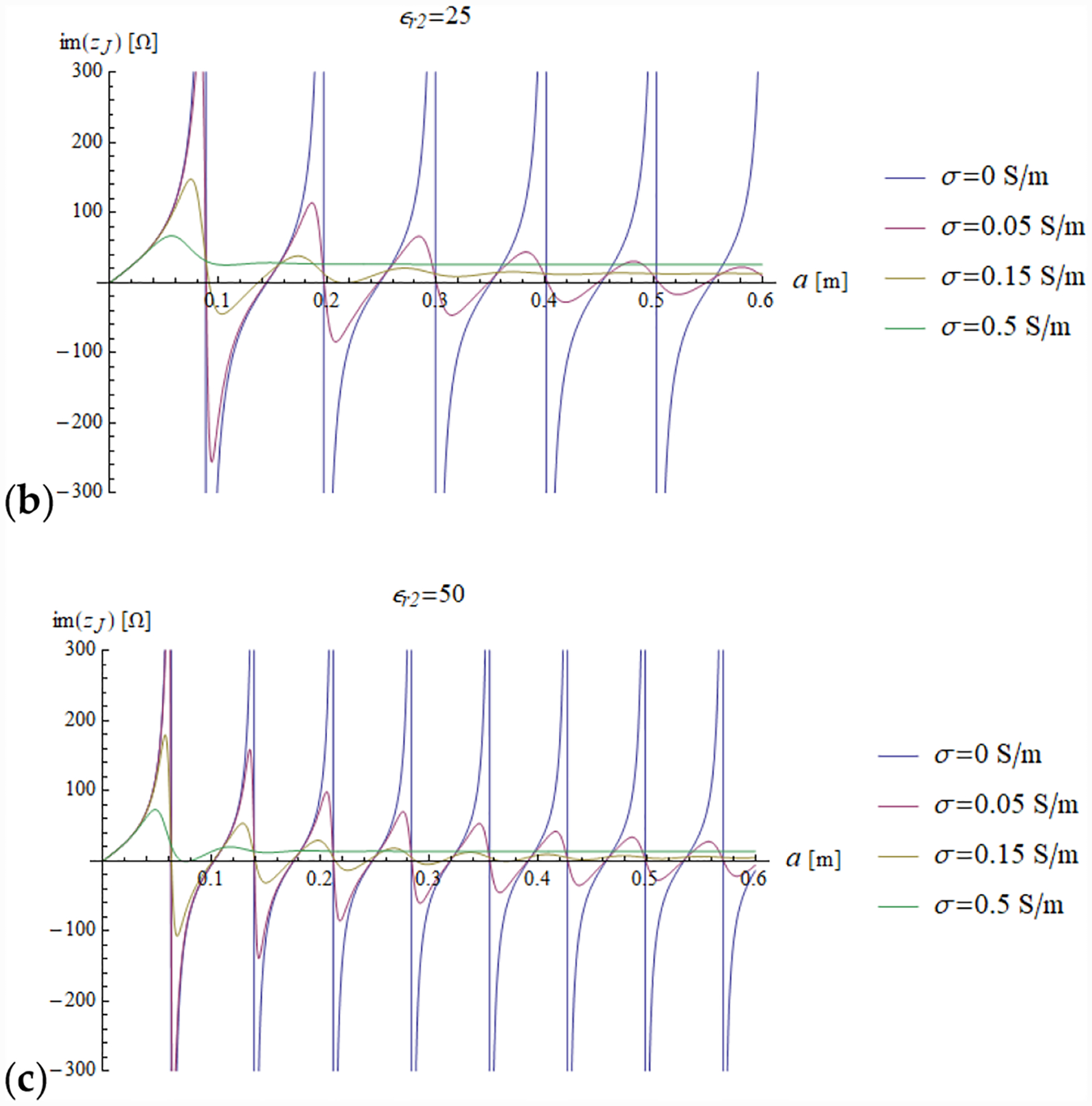
The imaginary part of the sphere impedance is plotted as a function of the sphere radius for different values of relative permittivity (*ε*_*r*2_ = 5, 25 and 50 in (**a–c**), respectively) and a range of conductivity values.

**Figure 6.**
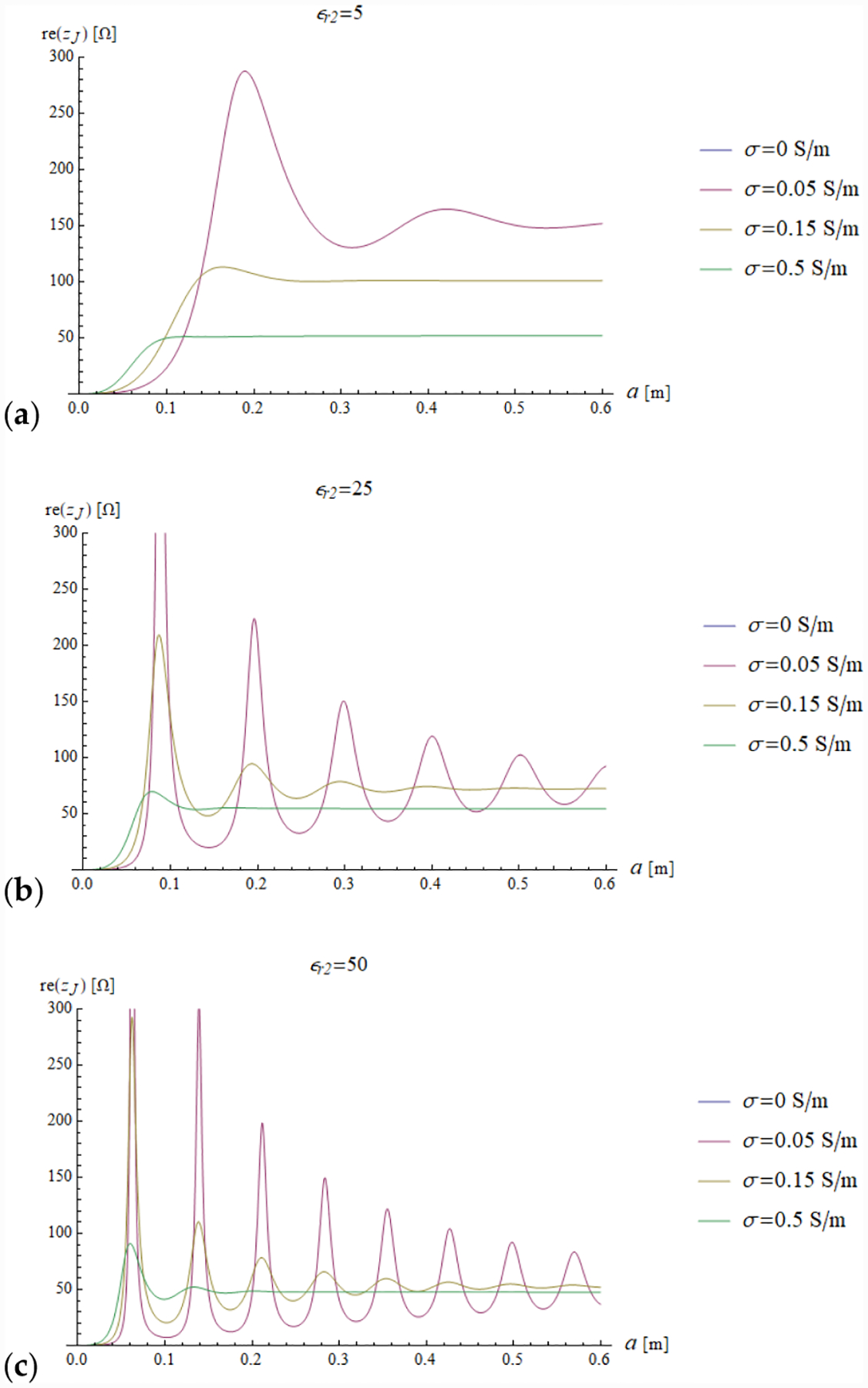
The real part of the sphere impedance is plotted as a function of the sphere radius for different values of relative permittivity *ε*_*r*2_ = 5, 25 and 50 in (**a–c**), respectively and a range of conductivity values.

In all the simulations, the carrier frequency was set to 292.7 MHz, which is the operating frequency of 7 T MR scanners, the external medium was air (*ε*_*r*1_ = 1) and the relevant quantities were plotted for a sphere with radius *a* ranging from 0 to 0.6 m (corresponding to *ka* = 26.93). We investigated the dependence of the reflection coefficient (Figure 4) and impedance (imaginary and real part in Figures 5 and 6, respectively) on different values of relative electric permittivity (*ε*_*r*2_ = 5, 25, 50) and conductivity (*σ* = 0, 0.05, 0.15 and 0.5 S/m).

**Figure 7.**
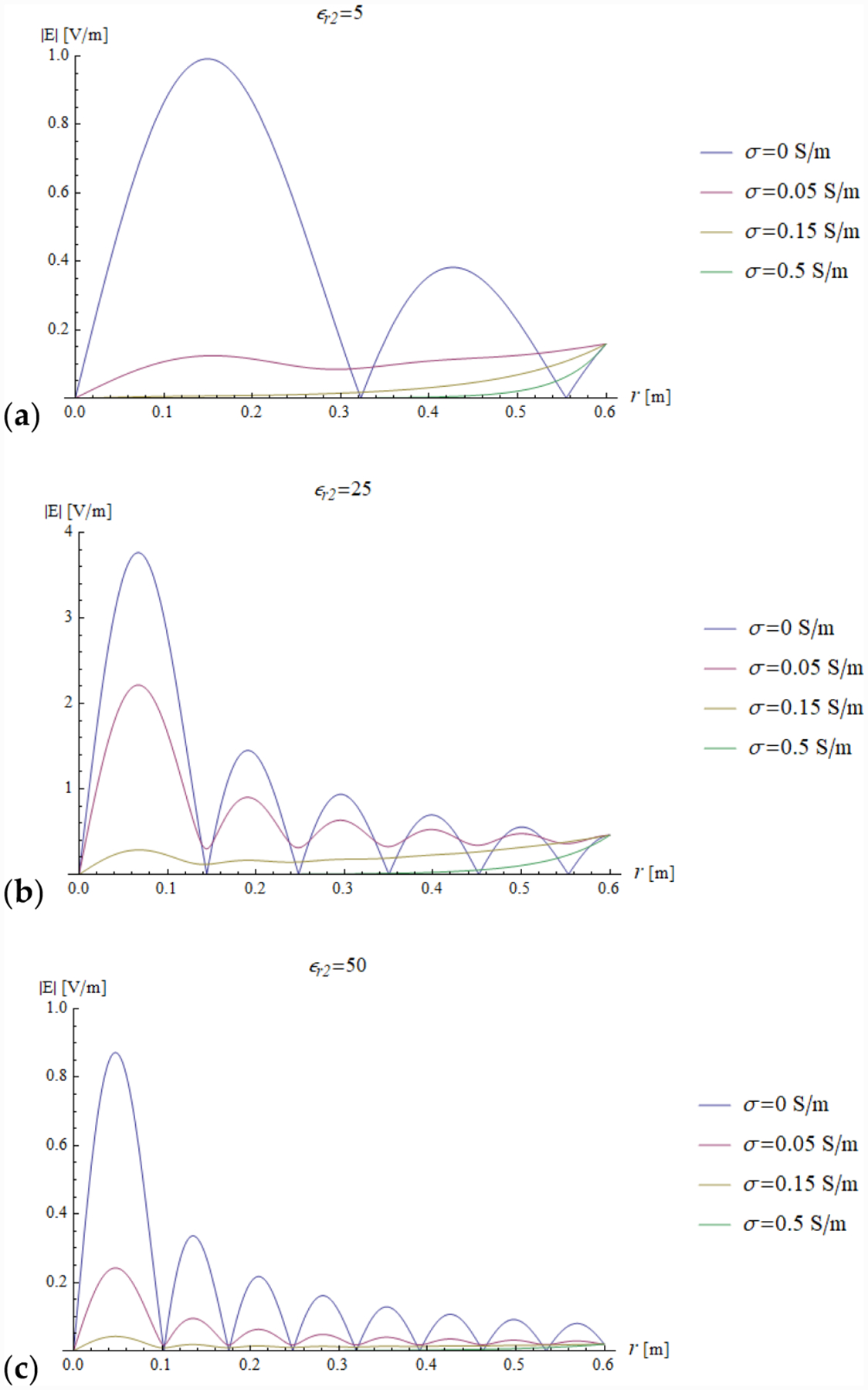
The amplitude of the electric field inside a sphere of radius *a* = 0.6 is plotted as a function of the radial coordinate r for different values of relative permittivity (*ε*_*r*2_ = 5, 25 and 50 in (**a–c**), respectively) and conductivity (see plot legend).

**Figure 8.**
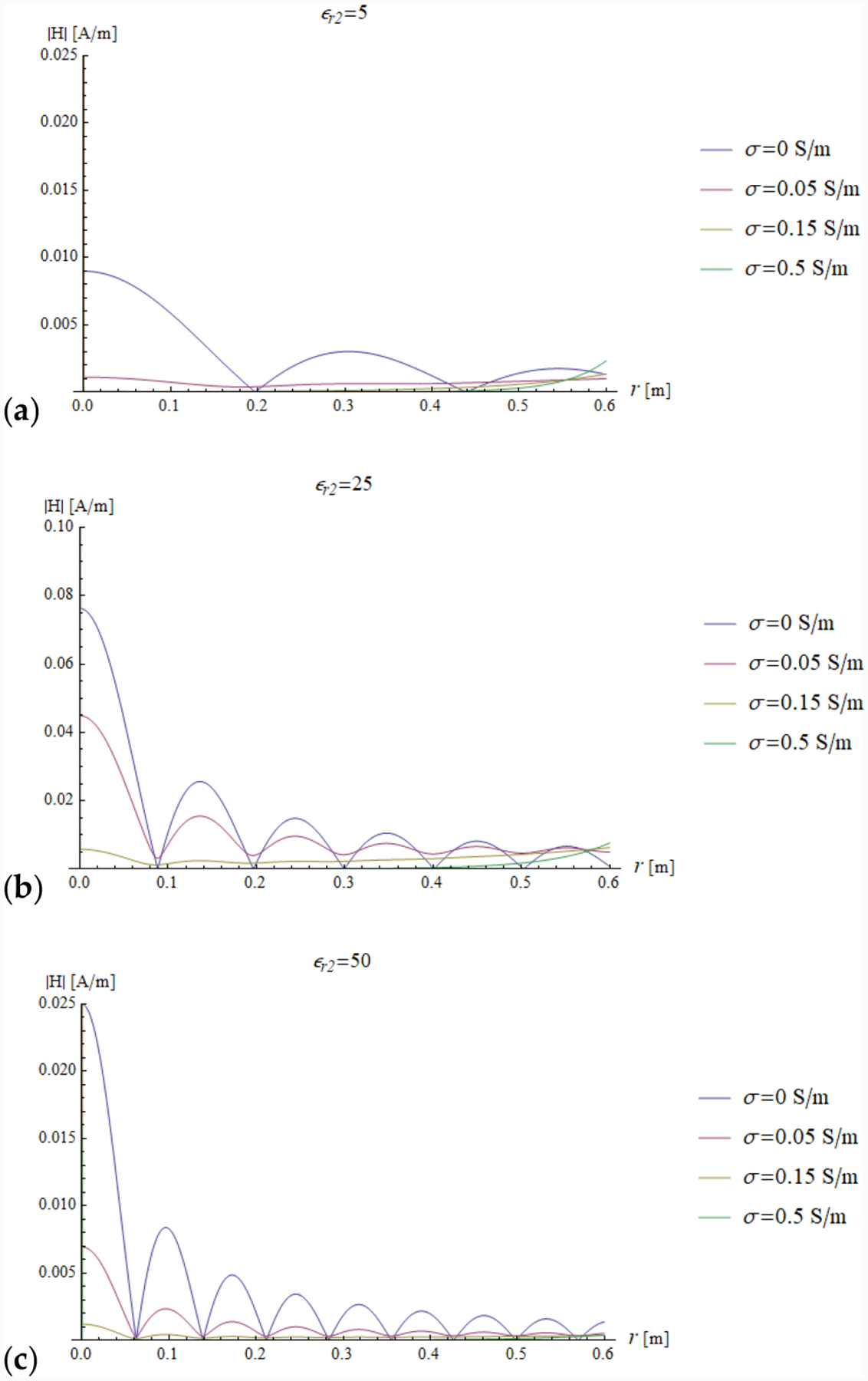
The amplitude of the magnetic field inside a sphere of radius *a* = 0.6 is plotted as a function of the radial coordinate r for different values of relative permittivity (*ε*_*r*2_ = 5, 25 and 50 in (**a**–**c**), respectively) and conductivity (see plot legend).

**Figure 9.**
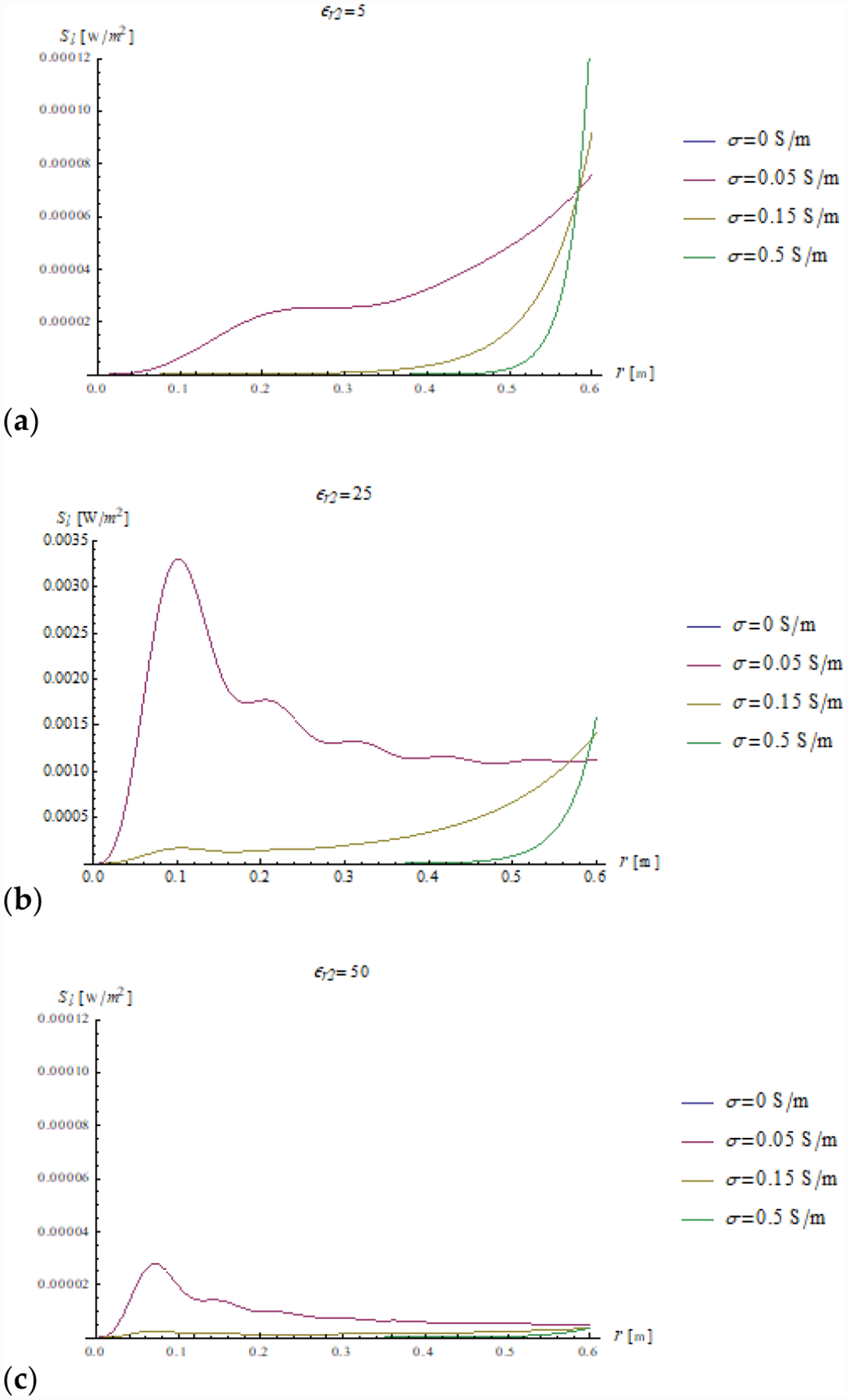
The amplitude is plotted as a function of the radial coordinate for different values of relative permittivity (*ε*_*r*2_ = 5, 25 and 50 in (**a**–**c**), respectively) and conductivity (see plot legend).

**Figure 10.**
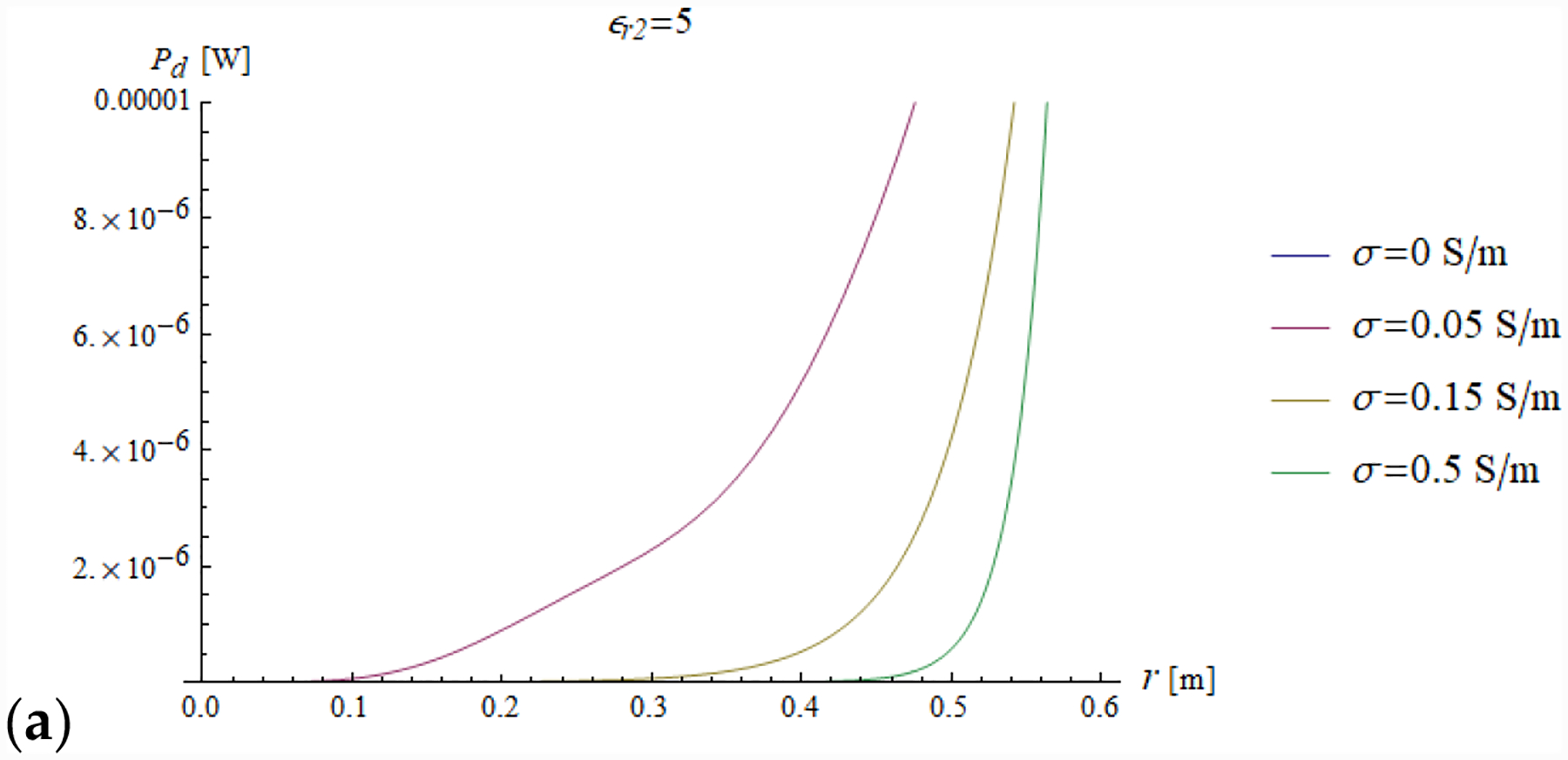

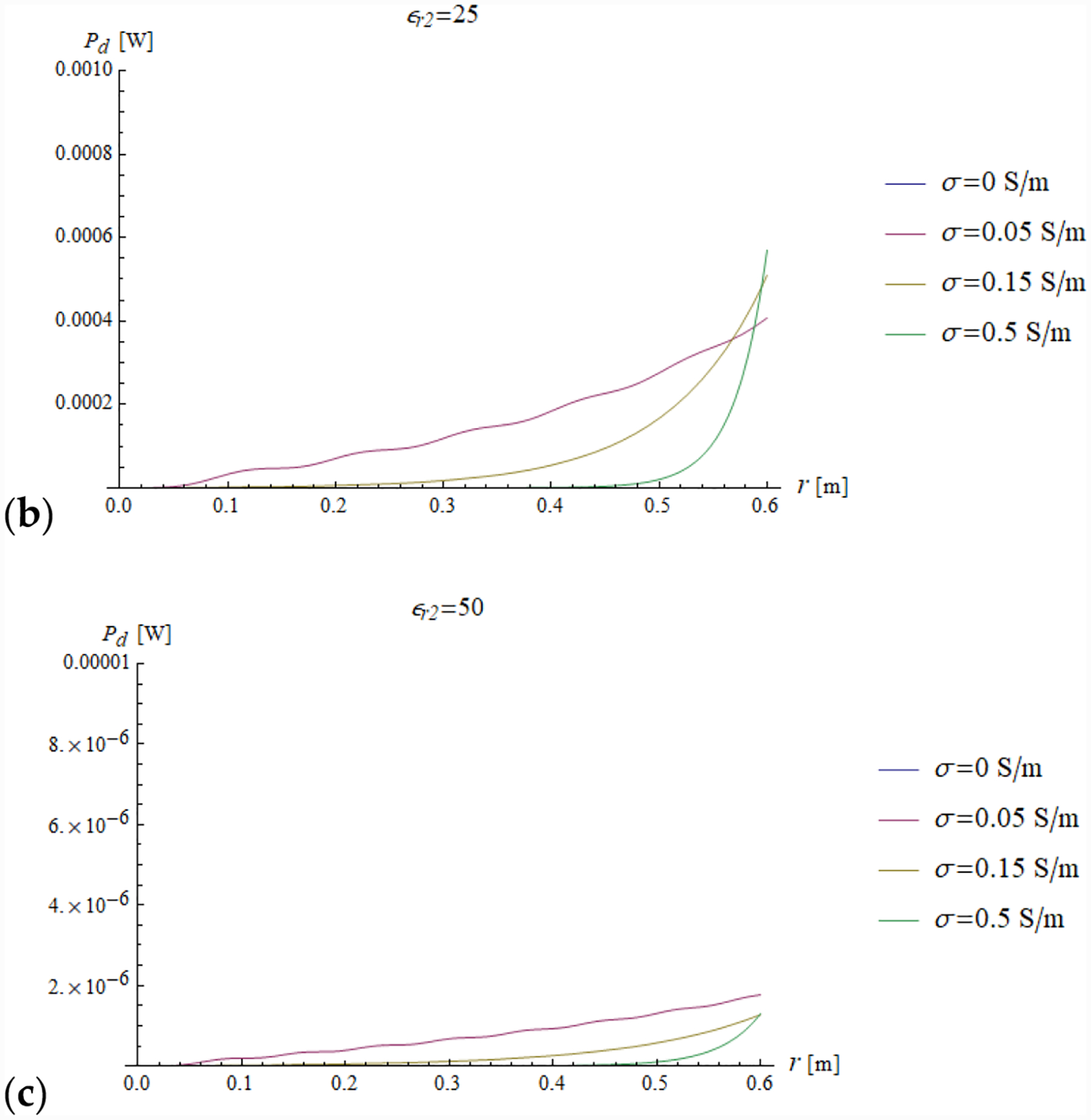
The power dissipated inside a sphere is plotted as a function of the radial coordinate for different values of relative permittivity (*ε*_*r*2_ = 5, 25 and 50 in (**a**–**c**), respectively) and conductivity (see plot legend).

In addition, we plotted the radial dependence of the EM field (electric and magnetic field in Figures 7 and 8, respectively) and the power (Figures 9 and 10) inside a sphere of radius *a* = 0.6 m for the same values of relative electric permittivity (*ε*_*r*2_ = 5, 25, 50) and conductivity (*σ* = 0, 0.05, 0.15 and 0.5 S/m).

The results for a lossless sphere (*σ* = 0 S/m) are shown in blue in all plots. For a lossless sphere, Figure 4 shows that the reflection coefficient has unitary modulus, independently from the value of the dielectric constant.

This is consistent with the fact that no power is absorbed by the sphere and all the incoming power (carried by inward waves) is balanced by the outgoing power (carried by outward waves). The same result is confirmed by the fact that the sphere impedance turns out to be a pure imaginary quantity.

In the following paragraphs, we show that our approach allows one to reformulate, in terms of engineering quantities (impedances and reflection coefficients), two typical boundary-value problems described in the literature by Mie scattering: the natural oscillation modes of a sphere and the diffraction of a plane wave by a sphere [2].

It is known from the Mie scattering theory that a lossless sphere is characterized by its natural modes that obey the transcendental equations introduced in Section 9.22 of [2] (Equations (10) and (19)). With our proposed approach, the same solutions are obtained simply by imposing the continuity condition for the impedance at *r* = *a*: *Z*_*n*_(*k*_1_*a*) = *Z*_*n*_(*k*_2_*a*).

By simply observing that in the inner medium there is a standing wave and that in the outer medium there is only an outward wave, it is straightforward to write the continuity condition by substituting to *Z*_*n*_(*k*_1_*a*) and *Z*_*n*_(*k*_2_*a*) the impedance expressions reported in the second and third rows of Table 1, respectively.

As a result, the condition can be simply written as:

(43) $$\bar{Z_{nl}}=Z_{Jn2}$$

or substituting the explicit expression reported in the second column of Table 1 as:

(44) $$\frac{1}{k_{1}}\frac{h_{n}^{\left(2\right)}\left(k_{1}a\right)}{h_{n}^{(2{)}^{\prime }}\left(k_{1}a\right)}=\frac{1}{k_{2}}\frac{j_{n}\left(k_{2}a\right)}{j_{n}^{\prime }\left(k_{2}a\right)}$$

Equation (43) provides the transcendental equation introduced in Section 9.22 of [2], whose solutions provide the natural frequencies of the sphere, demonstrating that a set of natural modes exists.

The main advantages of using our formulation are:

1. By employing the concept of impedance, we derived Equation (44) by means of simple and easily interpretable physical arguments.
2. Equation (44) can be easily extended to the case of a stratified sphere, which represents a considerable simplification compared to the classical approach that requires a complete reformulation of the scattering problem for each layer.

The diffraction of EM energy from a sphere can also be analyzed using our proposed framework. It is known that, when a plane wave impinges on a homogeneous lossless dielectric sphere, the EM field transmitted in the sphere (whose analytical expression is provided in Equation (33)) has a resonance-like behavior, governed by the geometric and dielectric characteristics of the sphere. From Equation (33), we can easily find the resonance condition by maximizing the ratio between the inward field coefficients of the two media, which occurs when the 1 + Γ_*n*_(*k*_1_*a*) factor reaches its maximum value, i.e., when the value of Γ*n*(*k*_1_*a*) is real and positive. Therefore, our formulation intuitively shows that resonances occur when outward and inward waves have electric fields in phase and magnetic fields in antiphase on the sphere surface. This means that the magnetic field is null on the sphere surface, which is consistent with the fact that the impedance *Z*_*Jn*_2(*k*_2_*a*) is infinite in resonance condition.

In perfect analogy with uniform transmission lines, our formulation allows one to interpret the resonance phenomenon in terms of the sphere’s impedance and, therefore, in terms of the phase difference between electric and magnetic fields on the spherical interace, which is responsible for the field distribution outside and inside the sphere and for the consequent energy storage.

The previous phenomena can be appreciated also directly from the plots of the impedance and the EM field. For example, a resonance effect can be seen in Figures 7b and 8b, where the maximum values of the amplitude of the electric and magnetic fields for *ε*_*r*2_ = 25 are considerably higher than the corresponding values for *ε*_*r*2_ = 5 (Figures 7a and 8a) and *ε*_*r*2_ = 50 (Figures 7c and 8c). For the fundamental mode (*n* = 1), for *a* = 0.6 m and *ε*_*r*2_ = 25, the product *ka* = 18.67 almost coincides with the sixth null of the derivative of the spherical Bessel function (18.79). From Figure 5 (see above) and Equation (31), we know that this corresponds to infinite impedance at the sphere surface, which means that the amplitude of the 1 + Γ_*n*_(*k*_1_*a*) factor is maximum and, therefore, the mode resonates.

In a similar manner, also in the TM case, we can obtain discrete resonance values corresponding to infinite values of the admittance at the interface, by looking at the nulls of the impedance at the interface.

For the case of a lossy sphere, the field is attenuated during its propagation and the inward–outward interferences are damped.

It is interesting to note that in this case the real part of the impedance is non-null, with peaks in correspondence of the resonant frequencies. In fact, the sphere impedance approaches the intrinsic impedance of the external medium, resulting in a minimum of the reflection coefficient (see Equation (31)).

Using our proposed framework, phenomena like those described above can be interpreted with traditional engineering concepts, such as an impedance pseudo-matching (in fact, perfect matching is not possible for a single sphere with real frequencies).

Relevant insight can also be gained from the power density plots. In a lossless sphere, the net incoming power density is fully balanced by the outgoing one and the net flux is null for all permittivity values (see Figures 9 and 10, where the blue line is superimposed to the x axis). This phenomenon is also shown by Equations (34) and (35), where it is clear that the net power density is null if the magnitude of the reflection coefficient ∣Γ_*n*_(*k*_*l*_*r*)∣ is unitary.

In the presence of losses, the power density closely follows the square of the amplitude of the electric field, and when *ε*_*r*2_ = 25, which is the in-resonance condition for the fundamental mode, for low losses (*σ* = 0.05 S/m), the power density is also large in proximity of the origin. If the losses are significant (*σ* = 0.15 and *σ* = 0.5 S/m), the power is mostly dissipated by the peripheral region of the sphere and the power density inside the sphere is almost null.

Figure 10 shows the power dissipation, which is the integral of the power density and is a monotonic function of the radial coordinate *r*. As expected, the higher the conductivity, the lower the capacity of the field to penetrate inside the sphere, and the power is mainly dissipated in the peripheral region of the sphere.

The power value at the boundary of the sphere (i.e., for *a* = 0.6 m in Figure 10) represents the total dissipated power inside the sphere.

It is worth highlighting that, with our formalism, the power was evaluated with a simple scalar expression (Equation (34)), without solving integrals, which represents an advantage compared to Mie scattering, in terms of the computation time and complexity associated with the integration of rapidly oscillating functions.

### 5.2. Full Modal Analysis

The behavior of the EM field for modes characterized by the higher order of the spherical Bessel functions was first investigated for the case of a lossless sphere.

**Figure 11.**
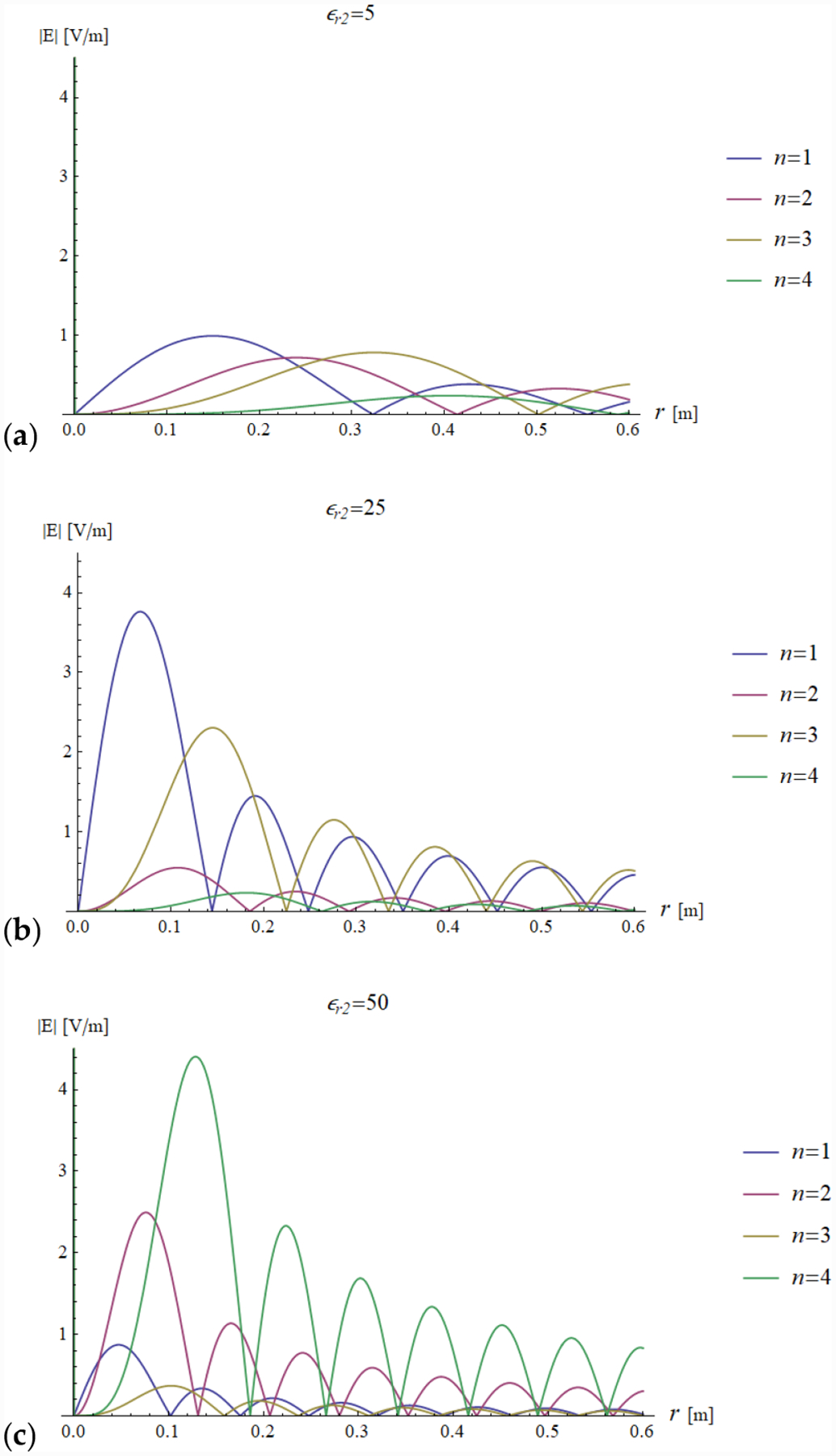
Electric field distribution as a function of the radial coordinate in a lossless (*σ* = 0 S/m) sphere of radius *a* = 0.6 m for the first four modes. Results are plotted for different values of relative permittivity (*ε*_*r*2_ = 5, 25 and 50 in (**a**–**c**), respectively).

**Figure 12.**
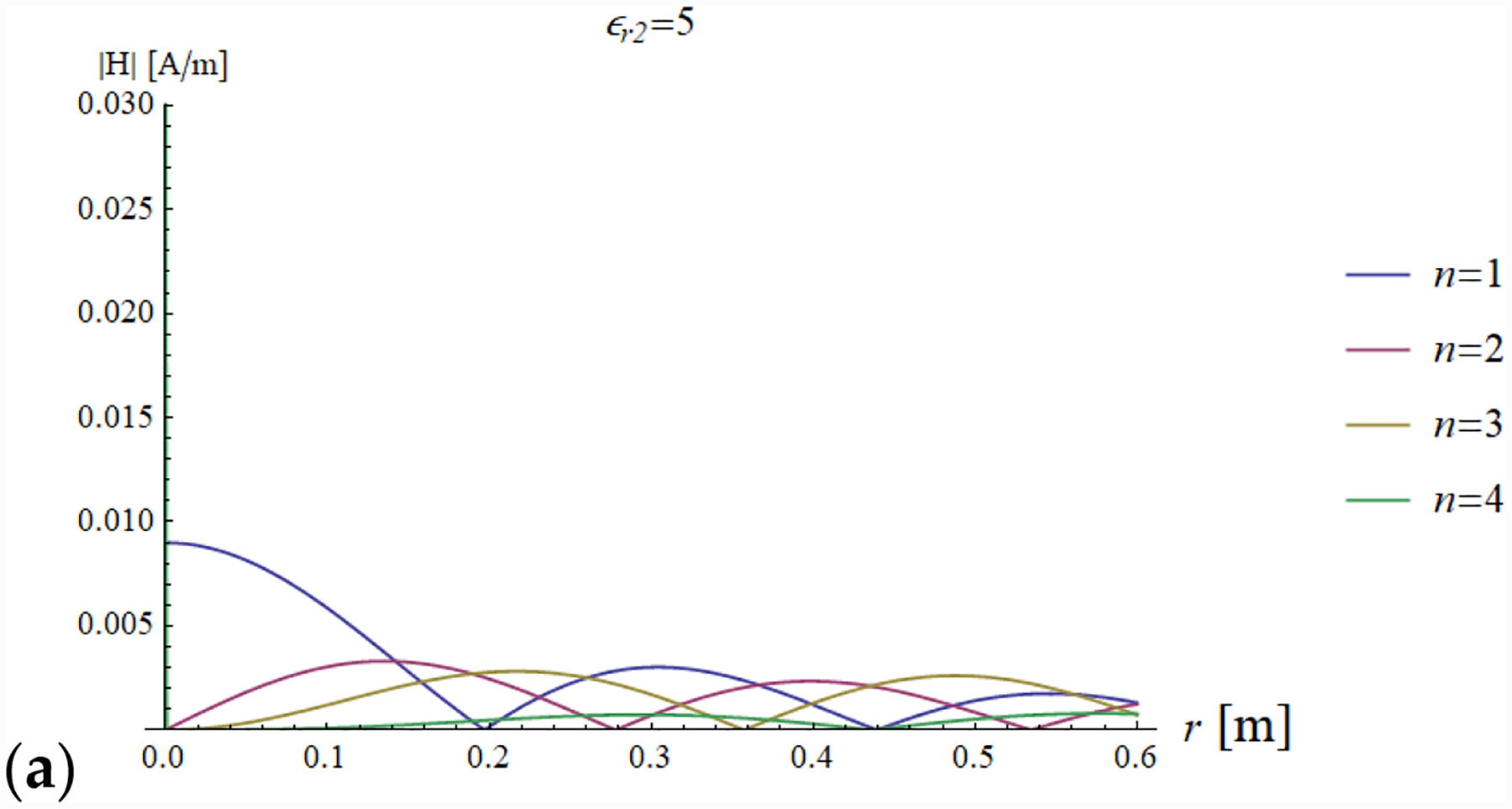

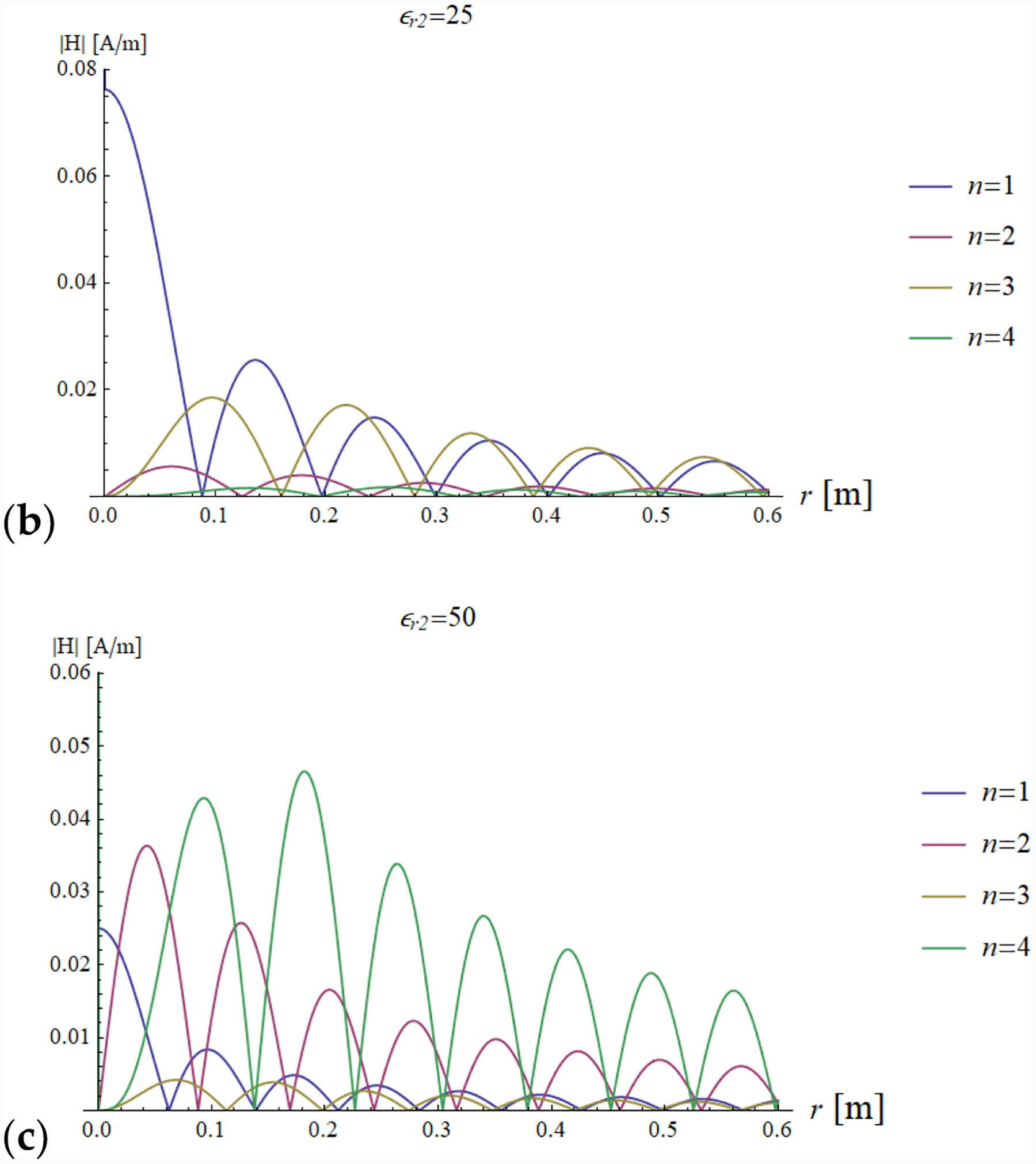
Magnetic field distribution as a function of the radial coordinate in a lossless (*σ* = 0 S/m) sphere of radius *a* = 0.6 m for the first four modes. Results are shown for different values relative permittivity (*ε*_*r*2_ = 5, 25 and 50 in (**a–c**), respectively).

Figures 11 and 12 show the transmitted electric and magnetic field as a function of the radial coordinate in a sphere of radius *a* = 0.6 m, evaluated for the first four modes of the Bessel functions.

For a low dielectric contrast (*ε*_*r*2_ = 5), none of the modes is resonant and the peaks of the fields are slightly decreasing with the order of the Bessel function. At the origin, the electric field is always null, due to field symmetry, and the only mode contributing to the magnetic field is the first mode.

Figure 11b,c and Figure 12b,c show that the field distribution is strongly influenced by the resonance conditions.

For example, as already noted when describing Figures 7b and 8b, when *ε*_*r*2_ = 25 and *a* = 0.6 m, the first mode is close to the resonance condition and, therefore, the corresponding electric and magnetic amplitudes both have a peak (Figures 11b and 12b). In addition, in the same figures, the third mode is also amplified, and this is due to the fact that *ka* = 18.67 is close to the fifth null of the derivative of the third order Bessel function, which corresponds to an infinite impedance and, therefore, to the resonance condition.

In Figures 11c and 12c, a similar pattern occurs for the second and fourth modes, which are on resonance when *ε*_*r*2_ = 50 and *a* = 0.6 m, which means they are the main contributors (among the first 4 modes analyzed in the plots) to the electric and magnetic fields.

A possible application of these results would be in the optimization of computation time. In fact, using the interpretation provided above, it is straightforward to order the modes based on their contribution to either the electric or magnetic field, and perform calculations only for the limited set of modes that contributes the most. In addition, the proposed scattering formulation in terms of engineering quantities could be useful for design optimization. For example, in MRI, it could guide the design of novel materials to be integrated with the radiofrequency coils ([30]) that can force impedance matching only for specific modes, in order to maximize the magnetic field (i.e., the source of the MRI signal) in a specific region while limiting power deposition (i.e., losses) over the entire sample [24].

**Figure 13.**
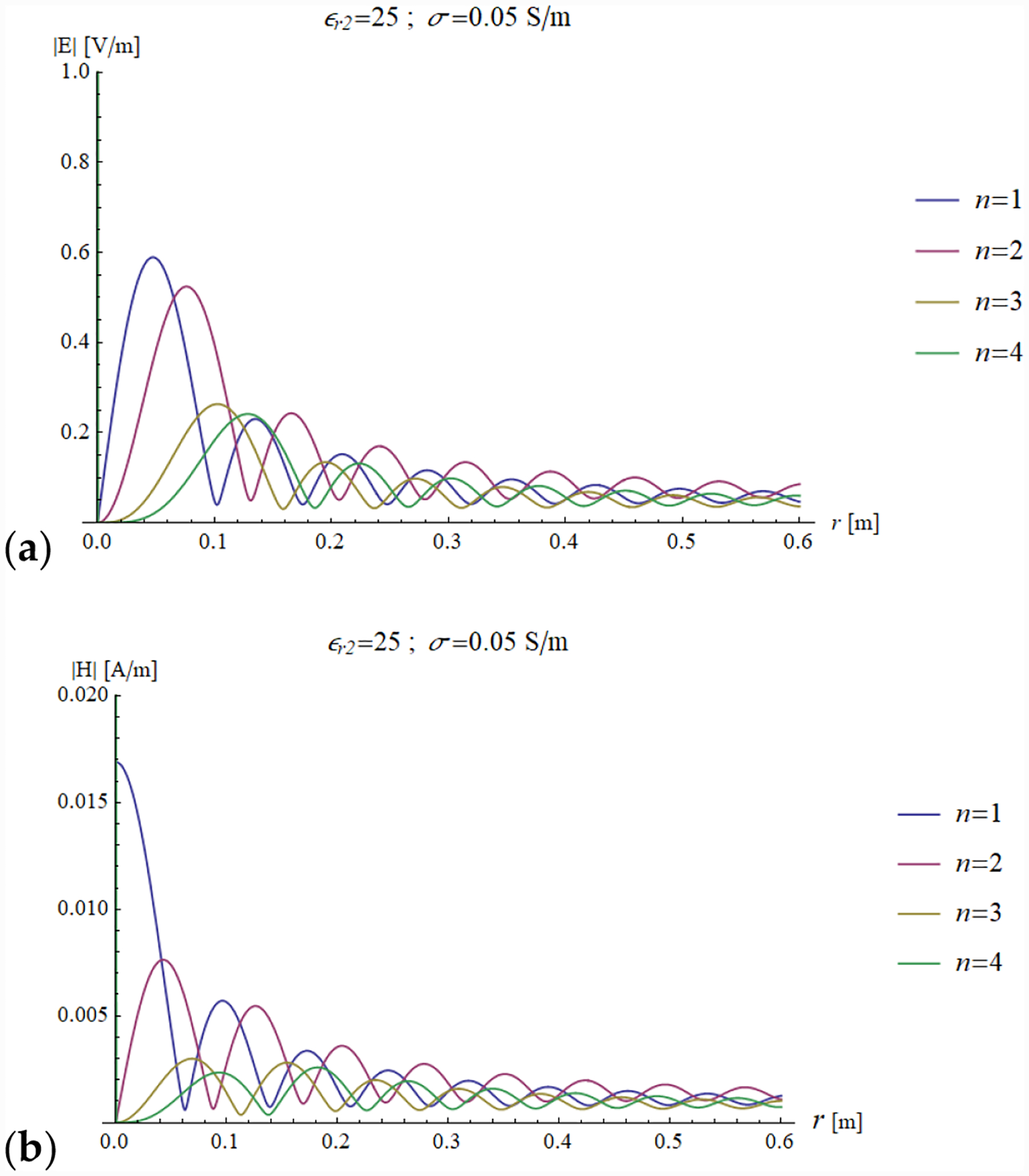
Electric (**a**) and magnetic (**b**) field distribution as a function of the radial coordinate for the first four modes. The sphere has a radius of *a* = 0.6 m, conductivity of 0.05 S/m and a relative permittivity of 50, mimicking the electrical properties of average brain tissue at 297.2 MHz.

In Figure 13, we show the EM distribution in the case of a lossy sphere, with conductivity *σ* = 0.05 S/m. As expected, all the modes are attenuated by the lossy medium. In addition, the resonance phenomenon cannot take place as for the case of a lossless sphere, because here the impedance cannot be infinite (see Figure 4), which results in considerable amplitude damping of the resonant modes (see y-axis scale of Figure 12 vs. Figures 10 and 11).

## 6. Discussion and Conclusions

In this work, we presented a theoretical framework to study the electromagnetic scattering and propagation characteristics in spherical objects. We demonstrated the proposed approach for the simple case of a homogeneous sphere, showing that it is fully consistent with the established Mie scattering theory. Our formulation extends previous work on equivalent transmission lines and the main advantage over Mie scattering is the possibility to analyze the propagation of the EM field in terms of reflection and transmission coefficients, making the physical understanding of the field distribution more intuitive. This goal was achieved by describing the fields inside and outside the sphere as a superposition of inward and outward waves, and forcing the equality of the inward and outward field coefficients inside the sphere to respect the energy conservation principle.

The described approach can be directly applied to research problems that currently use Mie scattering. One example is the design of metasurfaces, which enable unconventional phenomena, such as perfect absorption, holography, electromagnetic invisibility and much more [10,31–33]. In such application, the Mie coefficients are combined with homogenization techniques to evaluate the electromagnetic response of an array of high permittivity dielectric spheres, deriving a surface impedance. A possible implication of this study could be the expression of the surface impedance in terms of the impedance of the individual dielectric spheres, with an improvement of the physical interpretation behind the surface design.

Our model could be generalized in a straightforward manner to describe the scattering by multi-layered spheres, which has applications in several fields. In fact, our formulation can be seen as an extension of the theory of spherical transmission lines [25]. In particular, while the concepts of impedance and reflection coefficients in the analysis of the scattering from spheres were previously described, the significant novelty of this work is the introduction of an intuitive impedance transfer formula that simplifies the definition of the boundary conditions between layers. A possible application of our comprehensive theoretical framework could be the optimization of the properties of high-permittivity coil substrates that are used to manipulate the EM field distribution to improve the diagnostic performance of MRI coils [24,30].

While the proposed formulation provides intuitive physical insight if the sources surround the spherical object, as in various biomedical applications, it is also applicable to the classical problem of scattering of plane waves from spheres. In fact, the formulation is valid for any source that can be expressed as a linear combination of spherical waves.

One limitation of our approach is its effectiveness when a large number of modes is needed to describe the total electromagnetic field. In fact, in such case, it could be challenging to intuitively grasp an overall physical interpretation of the scattering from the analysis of the individual modes. Nevertheless, the framework would still allow one to identify a few dominant modes for the case of interest and study their behavior first.

In conclusion, the proposed method allows for expressing the scattering from spheres in terms of relevant engineering entities, providing physicists and engineers with a new tool to interpret the Mie scattering mathematical results, and to design systems that involve spherical scatterers with a full physical comprehension of the underlying phenomena.

## Figures and Tables

**Table 1.** Definitions of the different wave impedances.

Spherical Bessel Function	Impedance Expression	Impedance Symbol	Compact Expression
$h_{n}^{\left(1\right)}\left(k_{l}r\right)$	$\frac{i\omega \mu }{k_{l}}\frac{h_{n}^{\left(1\right)}\left(k_{l}r\right)}{h_{n}^{(1)\prime }\left(k_{l}r\right)}$	$Z_{n}^{\left(1\right)}\left(k_{l}r\right)$	*Z*_*nl*_
$h_{n}^{\left(2\right)}\left(k_{l}r\right)$	$\frac{i\omega \mu }{k_{l}}\frac{h_{n}^{\left(2\right)}\left(k_{l}r\right)}{h_{n}^{(2)\prime }\left(k_{l}r\right)}$	$Z_{n}^{\left(2\right)}\left(k_{l}r\right)$	$\bar{Z_{nl}}$
*j*_*n*_ (*k*_*l*_*r*)	$\frac{i\omega \mu }{k_{l}}\frac{j_{n}\left(k_{l}r\right)}{j_{n}^{\prime }}$	$Z_{n}^{\left(J\right)}\left(k_{l}r\right)$	*Z*_*Jnl*_

**Table 2.** Summary of the formulas defining the proposed framework.

	Electromagnetic Field	Reflection Coefficient	Impedance
*Traveling form*	$E_{lnm}(r)=E_{lnm}^{+}h_{n}^{\left(1\right)}\left(k_{l}r\right)\left[1+\Gamma_{n}\left(k_{l}r\right)\right]$	$\Gamma_{n}\left(k_{l}r\right)=\frac{Z_{n}\left(k_{l}r\right)-Z_{nl}}{Z_{nl}-Z_{n}\left(k_{l}r\right)\frac{z_{nl}}{\bar{z_{nl}}}}$	$Z_{n}\left(k_{l}r\right)=Z_{nl}\frac{1+\Gamma_{n}\left(k_{l}r\right)}{1+\Gamma_{n}\left(k_{l}r\right)\frac{z_{nl}}{\bar{z_{nl}}}}$
	$H_{lnm}(r)=\frac{E_{\text{lnm}}^{+}}{Z_{nl}}h_{n}^{\left(1\right)}\left(k_{l}r\right)\left[1+\Gamma_{n}\left(k_{l}r\right)\frac{Z_{nl}}{\bar{Z_{nl}}}\right]$		
*Stationary form*	$E_{lnm}(r)=\left(E_{lnm}^{+}+E_{lnm}^{-}\right)2j_{n}\left(k_{l}r\right)+i\left(E_{lnm}^{+}-E_{lnm}^{-}\right)2y_{n}\left(k_{l}r\right)$	$\Gamma_{n}\left(k_{l}r\right)=\frac{Z_{n}\left(k_{l}r\right)-Z_{nl}}{Z_{nl}-Z_{n}\left(k_{l}r\right)\frac{z_{nl}}{z_{nl}}}$	$Z_{n}\left(k_{l}r\right)=Z_{Jnl}\frac{A_{0l}+it_{nl}}{A_{0l}+it_{nl}^{\prime }}$
	$H_{lnm}(r)=\frac{k_{l}}{i\omega \mu }\left(E_{lnm}^{+}+E_{lnm}^{-}\right)2j_{n}^{\prime }\left(k_{l}r\right)+i\left(E_{lnm}^{+}-E_{lnm}^{-}\right)2iy_{n}^{\prime }\left(k_{l}r\right)$		

## Appendix A

In this appendix, we present the algebraic steps necessary to obtain the classical Mie scattering formulation from the reflection coefficient introduced in this paper. By using Equation (18) in Equation (31), we can write the ratio between outward and inward coefficients in Medium 1 as:

(A1) $$\frac{E_{1nm}^{-}}{E_{1nm}^{+}}==\frac{\frac{1}{k_{2}}\frac{jn\left(k_{2}a\right)}{j_{n}^{\prime }\left(k_{2}a\right)}-\frac{1}{k_{1}}\frac{h_{n}^{\left(1\right)}\left(k_{1}a\right)}{h_{n}^{(1{)}^{\prime }}\left(k_{1}a\right)}}{\frac{1}{k_{1}}\frac{h_{n}^{\left(1\right)}\left(k_{1}a\right)}{h_{n}^{(1{)}^{\prime }}\left(k_{1}a\right)}-\frac{1}{k_{2}}\frac{jn\left(k_{2}a\right)}{j_{n}^{\prime }\left(k_{2}a\right)}R}\frac{h_{n}^{\left(1\right)}\left(k_{1}a\right)}{h_{n}^{\left(2\right)}\left(k_{1}a\right)}=\frac{k_{1}h_{n}^{(1{)}^{\prime }}\left(k_{1}a\right)j_{n}\left(k_{2}a\right)-k_{2}j_{n}^{\prime }\left(k_{2a}\right)h_{n}^{\left(1\right)}\left(k_{1}a\right)}{k_{2}j_{n}^{\prime }\left(k_{2a}\right)h_{n}^{\left(1\right)}\left(k_{1}a\right)-k_{1}h_{n}^{(1{)}^{\prime }}\left(k_{1}a\right)j_{n}\left(k_{2}a\right)R}\frac{h_{n}^{\left(1\right)}\left(k_{1}a\right)}{h_{n}^{\left(2\right)}\left(k_{1}a\right)}$$

where the term *R* is defined as:

(A2) $$R=\frac{h_{n}^{\left(1\right)}\left(k_{1}a\right)}{h_{n}^{\left(2\right)}\left(k_{1}a\right)}\frac{h_{n}^{(2{)}^{\prime }}\left(k_{1}a\right)}{h_{n}^{(1{)}^{\prime }}\left(k_{1}a\right)}$$

Substituting Equation (A2) in Equation (A1), we obtain:

(A3) $$\frac{E_{1nm}^{-}}{E_{1nm}^{+}}=\frac{h_{n}^{(1{)}^{\prime }}\left(k_{1}a\right)j_{n}\left(k_{2}a\right)-\chi j_{n}^{\prime }\left(k_{2a}\right)h_{n}^{\left(1\right)}\left(k_{1}a\right)}{\chi j_{n}^{\prime }\left(k_{2}a\right)h^{\left(2\right)}\left(k_{1}a\right)-h_{n}^{(2{)}^{\prime }}\left(k_{1}a\right)j_{n}\left(k_{2}a\right)}$$

Using simple algebra, we can then derive the following quantity:

(A4) $$\frac{1}{2}\left(\frac{E_{1nm}^{-}}{E_{1nm}^{+}}-1\right)=\frac{j_{n}^{\prime }\left(k_{1}a\right)j_{n}\left(k_{2}a\right)-\chi j_{n}^{\prime }\left(k_{2}a\right)j_{n}\left(k_{1}a\right)}{\chi j_{n}^{\prime }\left(k_{2}a\right)h^{\left(2\right)}\left(k_{1}a\right)-{\dot{h}}^{\left(2\right)}\left(k_{1}a\right)j_{n}\left(k_{2}a\right)}$$

The left side of Equation (A4) is the ratio between scattered and incident fields expressed with the proposed formalism. The right side of the equation shows that this ratio has the same analytical expression of the TE Mie scattering coefficients presented in Equation (9), confirming that our proposed method is equivalent to the Mie approach.
